# Water, sanitation, hygiene practices, health and nutritional status among children before and during the COVID-19 pandemic: longitudinal evidence from remote areas of Dailekh and Achham districts in Nepal

**DOI:** 10.1186/s12889-022-14346-8

**Published:** 2022-11-07

**Authors:** Akina Shrestha, Bal Mukunda Kunwar, Regula Meierhofer

**Affiliations:** 1 Eawag, Swiss Federal Institute of Aquatic Science and Technology, Überlandstrasse 133, 8600 Dübendorf, Switzerland; 2 Kathmandu University School of Medical Sciences, Dhulikhel Hospital, Kathmandu, GPO Box 11008, Nepal; 3 Swiss Development Organization, Helvetas, Lalitpur GPO Box 688, Sanepa, Kathmandu, Nepal

**Keywords:** COVID-19, Water, Sanitation, Hygiene, Child health, Nutritional status, Problems due to COVID-19, Achham and Dailekh districts Nepal

## Abstract

**Background:**

The COVID-19 pandemic drew hygiene to the center of disease prevention. The provision of adequate water, sanitation, and hygiene (WASH) services is crucial to protect public health during a pandemic. Yet, access to levels of water supply that support adequate hygiene measures are deficient in many areas in Nepal. We examined WASH practices and their impact on child health and nutritional status in two districts before and during the COVID-19 pandemic.

**Methods:**

A longitudinal and mixed method study was conducted in March–May 2018 and November–December 2021. In total, 715 children aged 0–10 years were surveyed at baseline. Of these, 490 children were assessed at endline. Data collection methods included observations, a questionnaire, stool analysis, anthropometric measurements, water quality analysis, and an assessment of clinical signs of nutritional deficiencies. We conducted 10 in-depth interviews to understand major problems related to COVID-19.

**Results:**

Most respondents (94.2%) had heard about COVID-19; however, they did not wear face masks or comply with any social distancing protocols. Almost 94.2% of the households self-reported handwashing with soap 5–10 times per day at endline, especially after defecation, compared to 19.6% at baseline. Water quality was better at endline than at baseline with median 12 to 29 CFU *Escherichia coli*/100 mL (interquartile range at baseline [IQR] = 4–101) at the point of collection and 34 to 51.5 CFU *Escherichia coli*/100 mL (IQR = 8–194) at the point of consumption. Fever (41.1–16.8%; *p* = 0.01), respiratory illness (14.3–4.3%; *p* = 0.002), diarrhea (19.6–9.5%; *p* = 0.01), and *Giardia lamblia* infections (34.2–6.5%, *p =* 0.01) decreased at endline. In contrast, nutritional deficiencies such as bitot’s spots (26.7–40.2%; *p* = 0.01), pale conjunctiva (47.0–63.3%; *p* = 0.01), and dermatitis (64.8–81.4%; *p* = 0.01) increased at endline. The inadequacy of the harvest and the lack of household income to meet households’ nutritional needs increased drastically (35.0–94.2%; *p* = 0.01).

**Conclusion:**

We found that improved water quality and handwashing practices were associated with a decrease in infectious diseases. However, food security also decreased resulting in a high prevalence of nutritional deficiencies. Our findings underline that disaster preparedness should consider access to adequate WASH, nutrition, and health supplies.

**Supplementary Information:**

The online version contains supplementary material available at 10.1186/s12889-022-14346-8.

## Background

The corona virus disease of 2019 (COVID-19) was first detected in Wuhan (Hubei, China) in December 2019. After spreading globally, COVID-19 was declared a pandemic by the World Health Organization (WHO)[1–3]. Severe Acute Respiratory Syndrome Coronavirus-2 (SARS-CoV-2) was identified as the virus causing COVID-19 [4, 5]. The virus is transmitted primarily through the inhalation of respiratory droplets and aerosols and through contaminated fomites [6]. By March 2022, the WHO reported that COVID-19 had caused 6,029,852 deaths globally [7].

Even though most COVID-19 cases and deaths have been reported among adults, with many fewer cases among children [8], children in low- and middle-income countries (LMICs) may be more vulnerable to COVID-19, for several reasons [9]. First, children constitute a large proportion of the population of these countries. Second, risk factors for severe lower respiratory tract infection, such as exposure to smoke, malnutrition, and incomplete immunization, are more prevalent [10, 11]. Third, the burden of infectious diseases such as cholera and diarrhea is much higher among children (incidence of 528–629 per 1000 children with 36 per 1000 deaths [11]). Exposure to recurrent infections may weaken children’s immune systems, exposing them to higher risks of negative impacts of COVID-19 [12, 13]. In addition, recurrent diarrhea can negatively affect the intestinal microbiome, which is associated with a higher risk for children to suffer from severe COVID-19 and long COVID-19 [14]. Fourth, health systems in LMICs are under-resourced and weaker because the burden of adult COVID-19 has diverted resources away from child services, and this may further compromise child health [11]. Fifth, the scarcity of water and poor water quality and sanitation may promote the transmission of SARS-CoV-2 [15]. In addition, the indirect effects of the pandemic, including adult illnesses, unemployment, increasing poverty, and school closures, may lead to negative effects on child health and wellbeing [11].

Adequate access to water, sanitation, and hygiene (WASH) is crucial for protecting human health during outbreaks of infectious diseases[1, 16–18]. It is reported that the SARS-CoV-2 virus has also been detected in the feces of infected individuals. In LMICs with high rates of open defecation, ineffectual fecal sludge management, and poor access to safe drinking water, fecal–oral transmission may play a role in the virus transmission [19, 20]. Therefore, the WHO strongly advocates hand hygiene as a critical control measure to contain the transmission of SARS-CoV-2 [1, 16, 21]. WASH measures, such as proper handwashing with soap, could interrupt the transmission of diseases caused by bacteria and viruses [1]. Consequently, hygiene measures including regular handwashing with soap, regular hand disinfection, and safe disposal of feces have been promoted as measures for preventing the transmission of SARS-CoV-2 [21]. Even though several studies have highlighted the critical importance of WASH for preventing fecal–oral transmission of infectious diseases, the spread of SARS-CoV-2 via this route has not been confirmed [1, 22, 23]. Yet, the WASH sector in LMICs is attributed low priority and is underfunded despite of its critical importance to the control of infectious diseases, including COVID-19. As a result, a majority of the world’s population residing in LMICs lack access to adequate WASH facilities [11, 21, 24–26].

Inadequate WASH, malnutrition and infectious diseases are intricately linked [27]. While malnutrition is directly associated with insufficient dietary intake, underlying contributing factors, such as lack of access to safe and adequate WASH, result in recurrent infectious diseases such as intestinal parasites, diarrhea and COVID-19 [28, 29]. The intestinal parasites interfere with the digestive process by inhibiting the absorption of nutrients leading to compromised immunity of the host [27]. Evidence suggests that the COVID-19 virus could also get transferred from one surface to another via contaminated hands [30, 31]. Even though a direct relationship of COVID-19 and malnutrition is not reported, the indirect linkages could be related to steep declines in household incomes and changes in the availability and affordability of nutritious foods leading to child malnutrition, especially wasting. One in ten deaths among children below 5 years in LMICs is attributable to severe wasting because wasted children are at increased risk of mortality from infectious diseases [32]. Hence, in order to protect public health during the outbreaks of infectious diseases including the pandemic COVID-19, provision of WASH services is crucial [33].

In Nepal, especially in rural and hard-to-reach settlements, the combination of poor WASH conditions and limited access to already overstretched health care systems may be a serious reason for apprehension during the outbreak of infectious diseases [1, 25, 26]. Although COVID-19 does not appear to affect children in Nepal severely, the indirect effects of the pandemic could be of great concern, for several reasons [34]. First, access to health care was restricted, especially among low-income groups, due to lockdowns during the pandemic [35, 36]. This negatively affected the general healthcare provided to children in Nepal and included disruption of the childhood immunization service in some periods during the pandemic [34]. Reduced mobility due to lockdowns, poverty, fear of infection with COVID-19, and reduced access to overloaded health care facilities may have led to delays in seeking care for sick children, resulting in more severe illnesses [35, 37]. Second, children’s learning and their mental health are directly affected by the closure of schools [35, 38–40]. Children living in remote areas and in low-income households lack access to internet learning resources and do not have equitable access to distance learning [19, 41]. Lastly, the repeated lockdowns throughout the country resulted in widespread unemployment and major impacts on the parental involvement in economic activity leading to food insecurities that could have further compromised child health by reducing access to sufficient and balanced nutrition [11].

Public health interventions such as hand hygiene, wearing of face masks, social distancing, identification and isolation of infected people, and contact tracing have been promoted as measures to mitigate the epidemic [42, 43]. However, many of these measures are difficult or even impossible to institute in Nepal [35]. For instance, running water is not easily accessed, thus posing a challenge to adequate handwashing. People live in extremely crowded houses, making social distancing difficult. And the lack of infrastructure poses a challenge to supplying the products required for preventative hygiene measures such as soap, water treatment products, sanitizers, and face masks. In addition, the dissemination of public health measures and recommendations is difficult in remote rural communities that are often without electricity and road systems. Furthermore, there is dearth of evidence related to COVID-19 and its impact on WASH conditions, nutrition provided to children, child health before and during the COVID-19 pandemic in the rural communities of Dailekh and Achham districts in western Nepal. Hence, the objectives of this study were (a) to understand the uptake of COVID-19-related public health measures and (b) to explore the impact of COVID-19-related changes in WASH practices and nutrition on children’s nutritional and health outcomes in remote communities in Dailekh and Achham districts in western Nepal.

We sought to shed light on the WASH practices of people at the margin of society who are extremely poor, live in very remote areas, do not have access to internet services, and therefore cannot be reached through online media. We evaluated WASH-related behaviors and child health before and during the pandemic and documented the challenges that may affect respondents’ ability to comply with recommended WASH measures. A mixed-methods approach was used to capture respondents’ experience immediately after a period when the Delta covariant of SARS-CoV-2 was widespread throughout Dailekh and Achham districts. The knowledge gained by this research could help in the development of WASH-related guidelines for controlling outbreaks of infectious diseases such as COVID-19 in hard-to-reach areas of Nepal and improve the resilience of marginalized communities to epidemic shocks.

## Methods

### Study site and justification of site selection

The study was conducted in the Dailekh and Achham districts of Western Nepal in the Karnali province. The sites were selected on the basis of the following criteria: (a) remote mountainous region; (b) limited access to household water treatment products at baseline; (c) piped water supply scheme available; and (d) inadequate hygiene conditions [29]. Most of the 55 rural wards in Dailekh are connected with road networks, but only 15 rural wards are accessible during the monsoon season (June–August). The main occupation of the majority of the population is subsistence agriculture. Due to the low level of agricultural production, the majority of the households face acute food shortages for a large part of the year [44]. The average family size is 5.4 [45]. While 73.0% of the men and boys aged five can read, only 53.0% of the girls and women can. The diseases most frequently reported to the district’s health offices are acute respiratory infections, chronic obstructive pulmonary disease, chronic diarrhea, and indigestion. Malnutrition and undernourishment are prevalent problems in Dailekh [44].

**Fig. 1:**
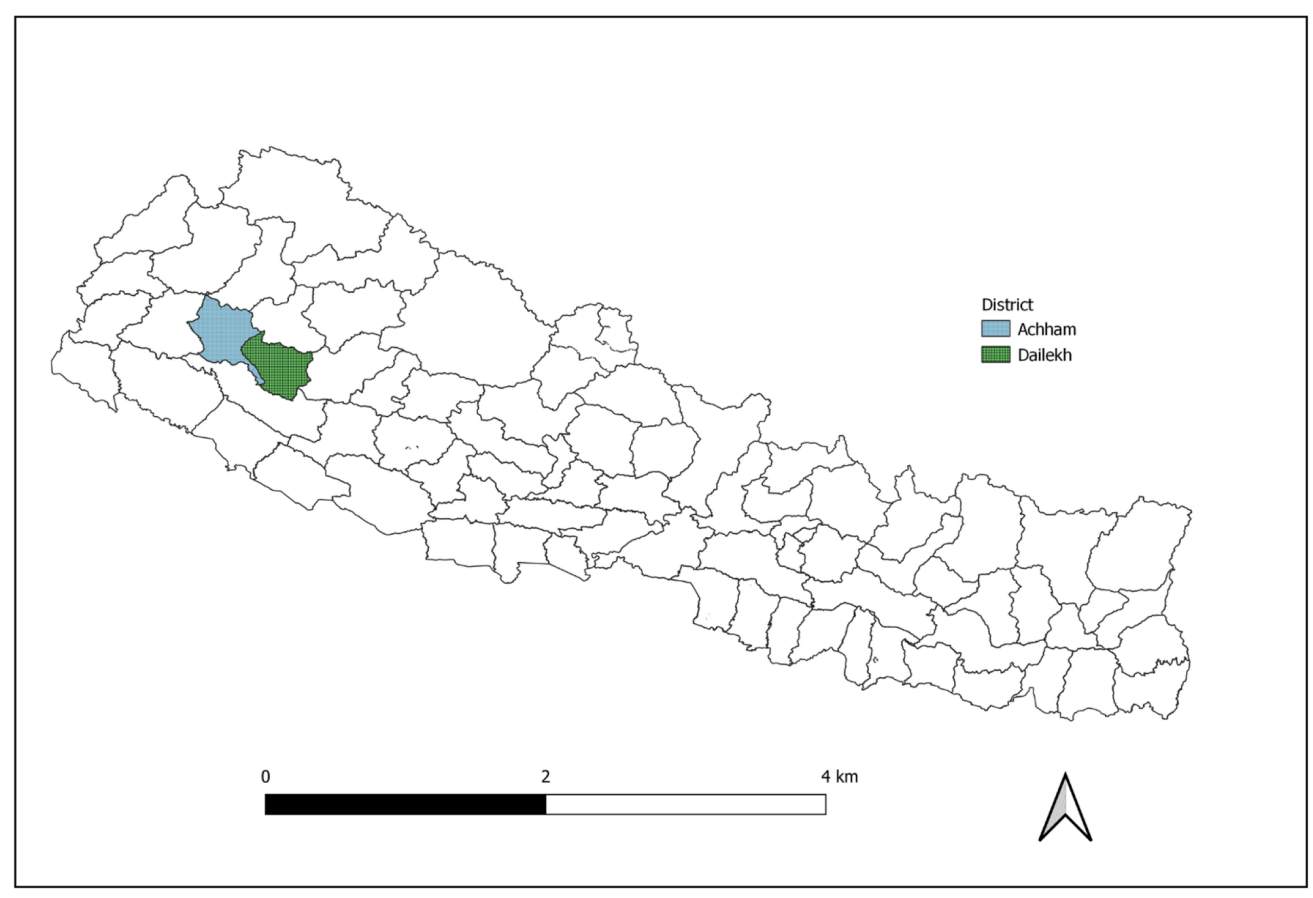
Map of Nepal showing study districts (Achham and Dailekh)

Achham is among the most remote and poorest districts in Nepal. It has 75 rural wards, of which 36 are connected with roads, although public transportation does not reach all of these. Dalits, the most discriminated caste, form about a quarter of the district’s population, and most of them live in poverty. About 71.0% of the men and boys aged five and above and 43.0% of the girls and women can read and write. Agriculture is the main occupation of the majority of the people in Achham. Work-related migration out of Achham is high. Male family members of at least one in three households, particularly from highly indebted Dalit and other socially excluded groups, practice seasonal migration to India. Most Dalits earn their living as daily wage laborers or masons or by making traditional products. The most common health problems reported at the district hospital are acute respiratory infections, scabies, diarrhea, gastritis, and uterus prolapse. Achham is among the districts with the highest prevalence of stunting, moderate acute malnutrition, and anemia in children under the age of five. The district is prone to natural disasters, such as floods, landslides, and forest fires [46]. A map of the study districts is shown in Fig. 1.

### Study design, sample population, sample size and sampling methods

Our study was originally designed as a randomized controlled trial with three WASH intervention arms and a control arm. Baseline data for the study were collected from March to May 2018 [29]. The endline survey was planned for March to May 2020. However, the endline data could not be collected at that time due to the emergence of the COVID-19 pandemic, national lockdowns, and travel restrictions in the project areas. Various WASH promotional messages were disseminated in the study areas during the pandemic through social mobilizers, female health community volunteers, media channels, and government and non-governmental organizations. In addition, water safety interventions were implemented, making it impossible to differentiate the impact of the two interventions. Therefore, endline data were collected during November and December 2021, after the wave of the Delta variant of the SARS-CoV-2 Virus. Because we are not able to differentiate between the interventions, we present aggregated longitudinal data of changes in WASH practices and nutrition on children’s nutritional and health outcomes in Dailekh and Achham districts of Nepal.

**Fig. 2:**
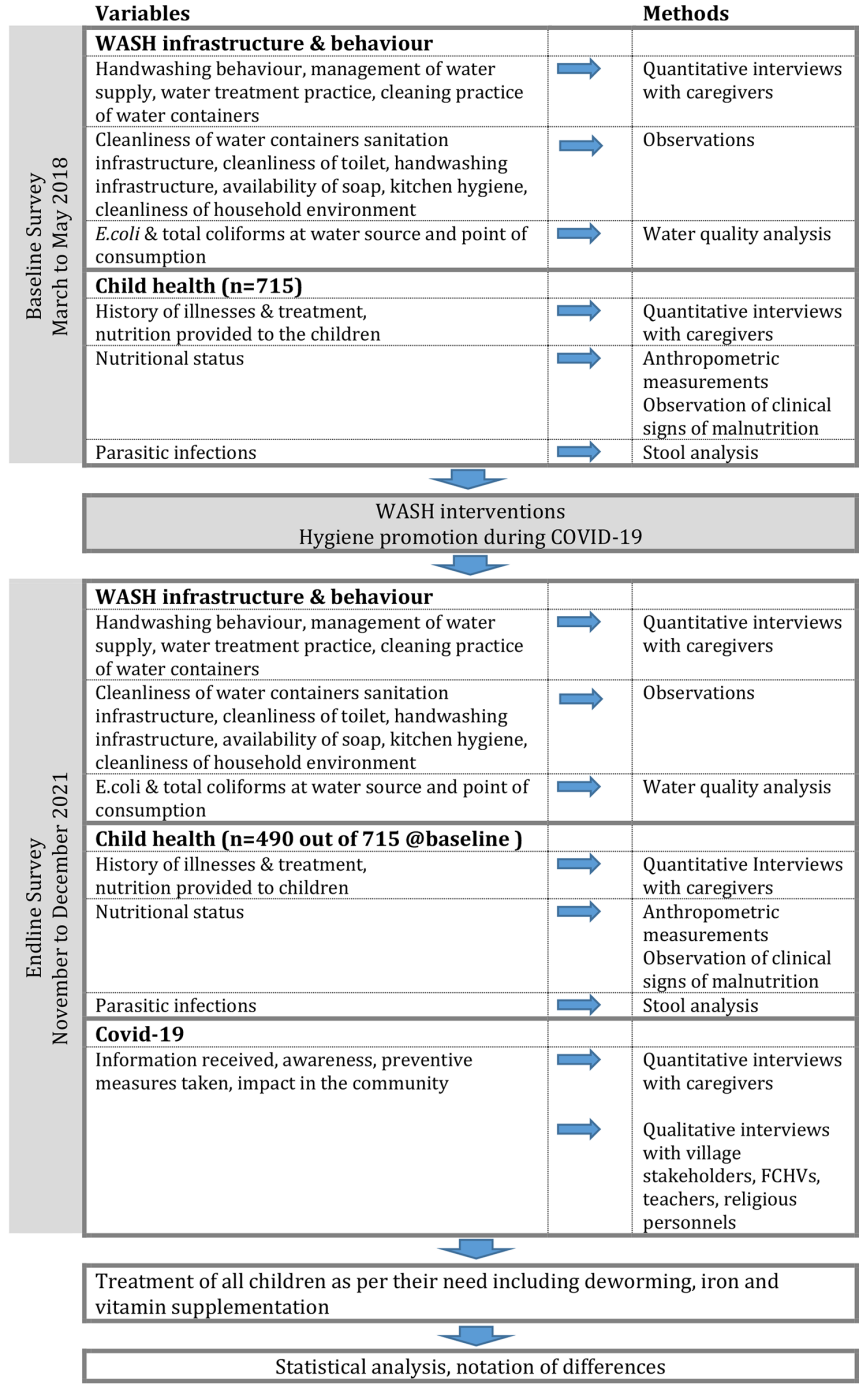
A schematic overview of the data collection methods of the study

Households with children aged 0–10 years were randomly selected at baseline, and 715 children were included. Of these, 490 children were reassessed at endline. The sample size and statistical powers were calculated with G* Power 3.1. A sample size of 300 households was required at each of the two sites at baseline to detect an effect in Cohen’s f2 with a one-tailed alpha of 0.05 and a statistical power of 90.0% with a mixed logistic regression and 15 predictor variables adjusting for the clustering effect of the study sites. The details of the sampling method have been published elsewhere [29]. A schematic overview of the data collection methods is provided in Fig. 2. Details of data and study variables are provided in Supplementary material: study variables 1.

### Quantitative interviews with children’s caregivers

Trained research assistants conducted semi-structured interviews with the caregivers of the same children, and the same questionnaire was used at baseline and endline. The semi-structured interview questionnaire included mostly structured questions and a few open-ended ones on water handling and hygiene practices, drinking water treatment availability and usage, sanitation, household cleaning practices, WASH infrastructure, and WASH promotion activities that they had received. Child dietary information was assessed following the guidelines of the Food and Agricultural Organization [47]. The caregivers were asked whether nine food groups were consumed within the past seven days and the frequency of consumption. Household food security was assessed using questions relating to the availability of adequate food during whole year. The interview tool also included specific questions on child health and nutritional status, following the international guidelines [47]. During the endline survey, additional questions were included on COVID-19 related information such as knowledge, attitude, and practices; symptoms and history of COVID-19; WASH measures adopted for the prevention of COVID-19 and related health-seeking behaviors; source of information about COVID-19; mitigation measures; and impact of the pandemic on their daily lives. The interviews were complemented by structured observations on the status of WASH infrastructure and hygiene in the households. The questionnaire was coded with Open Data Kit software on tablets (Samsung Galaxy note 10.1 N8010) and pretested and adapted to meet the conditions of the study sites.

### Qualitative interviews with village stakeholders

Qualitative semi-structured in-depth interviews were conducted by the first author during the endline survey to capture the respondents’ opinions on general problems and WASH practices relating to COVID-19. The interviews were conducted in various wards of Dailekh and Achham districts with teachers, health workers, and religious leaders. In addition, we conducted qualitative interviews with the children’s caregivers to obtain more detailed insights into the challenges they faced during the pandemic. Our analysis focused on the impact of COVID-19 on WASH practices, including drinking water treatment, handwashing practices, sanitation management, household hygiene, child health and nutritional status.

### Clinical signs of nutritional deficiency

Clinical signs of nutritional deficiencies were assessed by certified medical assistants at both baseline and endline using a standard checklist. The children were examined for (a) bitot’s spots, (b) loss of hair pigment, (c) dry and infected cornea, (d) oedema, (e) pale conjunctiva, (f) bowed legs, (g) spongy bleeding gums, (h) dermatitis, (i) red and inflamed tongue, (j) subdermal hemorrhage, and (k) goiter [48].

### Anthropometric measurements

The certified medical assistants also conducted anthropometric measurements of the children’s height and weight at both baseline and endline, adhering to standard operating protocols [49]. For children younger than 2 years, Seca Baby Mat 210 was used for supine lengths, and for children aged between 24 months and 10 years, a height-measuring board and a digital scale (Seca 877; Hamburg, Germany) were used [50]. In accordance with WHO guidelines, AnthroPlus (WHO; Geneva, Switzerland) was used to calculate anthropometric indices [49, 51]. The anthropometric indices expressed as z-scores were (a) height for age (HAZ, stunting); (b) weight for age (WAZ, underweight), and (c) body mass index for age (BAZ, thinness) [52]. Z-scores of $\ge$ -2 were regarded as normal, those between < 2 and ≥ -3 as moderate undernutrition, and those below < -3 as severe undernutrition [29].

### Parasitological survey

At both baseline and endline, the caregivers were asked to provide a fresh morning stool sample from the participating child on the day following the household survey. The samples were processed on the same day by an experienced laboratory technician using (a) duplicate Kato-Katz thick smear for helminths; (b) direct wet-mount; and (c) formalin-ether concentration for protozoa and helminths following standard guidelines [53–56]. The detection of one or more eggs of any worm species on either slide was defined as the presence of infection. The infection intensity of helminths was calculated according to criteria defined by the WHO [57].

### Drinking water quality examination

The drinking water samples were collected by the certified medical assistants from the household’s main drinking water source (point of collection) and from the container used for drinking water transport (point of consumption) at both baseline and endline. The water sample at the point of collection was collected from the tap after letting the water run for 60 s. The caregivers were asked to bring fresh drinking water from the main drinking water source to the household in the same container they normally use to collect drinking water. The water samples were then poured into the Naso Whirl Pak bags and immediately analyzed on site using the membrane filtration technique: 100 mL water samples were passed through 0.45 μm sterile millipore cellulose membrane filters with sterilized filtration equipment, and the filter pads were plated on Nissui Compact Dry Coliscan plates. The plates were then incubated for 24 h at 35 +/- 2 °C and *Escherichia coli* (*E. coli*) were counted after 24 h [29, 58].

### Data collection

The data was collected by a team of 12 enumerators: 6 trained research assistants for the quantitative interviews and observational checklists and 6 certified medical assistants for conducting children’s anthropometric measurements, examining clinical signs of nutritional deficiencies, and analyzing water quality. They were all fluent in the local language (Khas) of Dailekh and Achham districts. The team underwent four days of training in March 2018 and refresher training in November 2021 on how to conduct face-to-face interviews, including different interview techniques for structured and open-ended questions, collecting and analyzing water samples, conducting children’s anthropometric measurements, and collecting children’s stool samples.

Before data collection, the enumerators introduced themselves and the objective of the study and read a consent form which included consent to examine and measure the child and to observe drinking water collection at the point of collection and point of consumption. Respondents were also informed about the concept of voluntary participation. Once the respondent provided written informed consent, the research assistants proceeded with an interview. Each interview took approximately 20–30 min. The study protocol was approved by the Kantonale Ethikkommission, Zurich in Switzerland (KEK, reference no. 2018-00089) and by the Nepal Health Research Council, Kathmandu in Nepal (NHRC, reference no. 2956). The respondents were provided with an opportunity to decline or reschedule the interview or to withdraw from the interview whenever they chose. Further details of the selection of the respondents and of the data collection procedures have been reported elsewhere [29]. The interviewees for the qualitative interviews were purposively selected to include representatives from different sociodemographic backgrounds with differing influences on the community and permanent residence in it. The respondents were selected after consultation with two nongovernmental organizations: the Social Service Centre (SOSEC) and the Rural Development and Empowerment Center (RUDEC) Nepal, located in Dailekh and Achham districts, respectively. These organizations have been working on WASH promotion in the study areas over the last eight years. A topic guide, which was developed for the interview, was translated and pretested before the first interview. It covered experiences and problems faced during the COVID-19 pandemic regarding livelihood, family, WASH practices, health, education, and nutrition at the household and community levels. The first author visited the households of the selected respondents and personally conducted 10 in-depth interviews. Each interview began with an introduction to build rapport before the respondents were asked to share their experiences of COVID-19 during the pandemic and its impact on the lives of the children. Each interview lasted for 15 to 30 min.

### Data management and statistical analysis

The quantitative data collected was controlled daily, and if any values were missing or inconsistent, the households were revisited and consulted the following day. The readings of intestinal parasite and nutritional deficiency screenings were double-entered into an Excel 2010 spreadsheet (Microsoft; Redmond, USA) and cross-checked. Numerical variables were described by mean and standard deviations, if normally distributed, and by medians and interquartile ranges otherwise. Categorical variables were described by absolute and relative frequencies. The McNemar test for binary outcomes, Wilcoxon rank-sum test for continuous variables with non-normal distribution, and χ2 statistics were used to assess the differences in the distribution of categorical variables between the study areas at baseline and endline. The percentage of responses to the questions were calculated according to the number of respondents per response and the number of total responses to the questions and presented as categorical variables [59].

Principal component analysis (PCA) was used to characterize household socioeconomic status, and this was based on factor analysis of reported household assets: electricity, radio, TV, solar panel, mobile phone, bicycle, motorbike, fridge, watch, household ownership, number of household rooms, and landownership. Three factors reflecting three socioeconomic domains (low, medium, and high) were retained and divided, using the Kaiser Normalization (*k*-means) procedure. The same procedure was applied to create one variable for the cleanliness of containers used for latrine hygiene (kind of latrine, cleanliness, availability of sandals, water drum, and brush or the absence of these materials); cleanliness of the household environment (placement of garbage and garbage pit outside the households and cleanliness around the household); kitchen hygiene (dirty dishes, food covered, dry racks, and flies in the household); and personal hygiene (parents wearing shoes, and the cleanliness of the parents’ and children’s hands and children’s clothes). PCA was performed, using varimax rotation to maximize the sum of the variance of the factor coefficients. The number of factors chosen was based on *k*-means, where only factors with eigenvalues > 1.0 were considered [60].

To assess the change in prevalence, incidence, and persistence of health outcomes between baseline and endline, we only included children in the analysis for whom data from both the baseline and endline surveys were available (*n* = 490). Mixed logistic regression models with random intercepts of study wards, adjusting for age, sex, ethnicity, and socioeconomic status, were used to estimate the changes in incidence and persistence of binary health outcomes, such as fever, cough, respiration problems, diarrhea, blood and mucus in stool, blood in urine, and intestinal parasitic infection, including *Ascaris lumbricoides*, *Trichuris trichura*, *Hymenolepsis nana*, hookworm, *Enterobius vermicularis*, and *Giardia lamblia* [27]. To assess the changes in prevalence, generalized estimating equations for repeated measures analyses with random intercepts at the level of children were used. The change in prevalence was determined by persistence, for instance children who were wasted at baseline and were still wasted at endline, and incidence along with the baseline prevalence according to this formula:

Prevalence at endline = (prevalence at baseline) + prevalence + (1-prevalence at baseline) + incidence [27].

We used mixed linear regression models with random intercepts for study wards, adjusting for the age and sex of the children, the socioeconomic status of the caregivers and the districts to assess the longitudinal changes of continuous variables. These models included the baseline value of each outcome as one of the predictor variables along with age, sex, district, and socioeconomic status [27]. The changes were considered statistically significant if *p*-values were < 0.05. All analyses were carried out with STATA, version 14 (STATA Corporation; College Station, TX, USA).

The qualitative interviews were transcribed verbatim in Nepali and then translated into English for the analysis. The analysis then followed Graneheim and Lundman’s content analysis steps [61]. The interviews were read several times to obtain a sense of the whole, incorporating nonverbal communication noted during the interviews. Then, the meaning units were identified from the interviews. The meaning units were condensed and abstracted and labeled with codes. Subsequently, the codes were grouped in subcategories according to their similarities and differences. Authentic quotations are given below to support and confirm the findings and represent reality.

## Results

### Study compliance and characteristics of the study population

Of the 715 children who were enrolled during the March 2018 baseline survey, 486 children’s primary caregivers completed the questionnaire survey during the endline and 490 children completed all aspects of the health and nutritional examinations. During endline, 493 water samples from households’ main drinking water sources and 493 from the containers used for drinking water transport and storage were collected. Due to the COVID-19 pandemic and migration, 225 households were no longer accessible, 222 children included during the baseline could not be retraced, three children had died, and three caregivers were away and could not be interviewed. We compared the socioeconomic status of the households at baseline with that at endline. The number of households with high socioeconomic status increased slightly from 34.3 to 34.6%. The percentage of households with average socioeconomic status decreased from 33.3 to 32.7%, and households with low socioeconomic status remained almost constant from 32.5 to 32.7% over the 33-month study period.

The characteristics of the children and caregivers who completed the endline survey are described in Table 1. More than half of the children surveyed were female (55.5%). The primary occupation of the caregivers was in agriculture at both baseline (72.5%) and endline (78.0%). Almost 72.2% had domestic animals in their households during the endline survey. Most of the children’s households owned agricultural land at both baseline (96.2%) and endline (97.3%). The great majority (79.9%) of the households did not have electricity in their household. There was also no access to the internet and no road network in the study wards.

**Table 1:** Characteristics of the study population in Dailekh and Achham districts of Nepal, March-May 2018 and November to December 2021

Demographic and socioeconomic characteristics	Overall [N (%)]		Dailekh [n (%)]		Achham [n (%)]	
	Baseline	Endline	Baseline	Endline	Baseline	Endline
Caregivers socio-demographic characteristics						
Sex of the caregivers						
Female	653 (91.3)	423 (87.0)	332 (93.3)	216 (88.5)	321 (89.4)	207 (85.5)
Male	62 (8.7)	63 (13.0)	24 (6.7)	28 (11.5)	38 (10.6)	35 (14.5)
Age of the caregivers						
15–25 years	176 (24.6)	63 (13.0)	105 (29.5)	42 (17.2)	71 (19.8)	21 (8.7)
26–40 years	421 (58.9)	307 (63.2)	207 (58.1)	151 (61.9)	214 (59.6)	156 (64.5)
> 40 years	118 (16.5)	116 (23.8)	44 (12.4)	51 (20.9)	74 (20.6)	65 (26.8)
Caregiver’s literacy						
Can neither read or write	192 (26.8)	188 (38.7)	41 (11.5)	62 (25.4)	151 (42.0)	126 (52.1)
Can read only	15 (2.1)	15 (3.1)	14 (3.9)	13 (5.3)	1 (0.3)	2 (0.8)
Can both read or write	508 (71.1)	283 (58.2)	301 (84.6)	169 (69.3)	207 (57.7)	114 (47.1)
Highest education level caregivers have completed						
Informal education	327 (45.7)	128 (26.3)	109 (30.6)	41 (16.8)	218 (60.7)	87 (36.0)
Primary	154 (21.5)	156 (32.1)	101 (28.4)	62 (25.4)	53 (14.8)	94 (38.8)
Secondary	130 (18.2)	57 (11.7)	89 (25.0)	39 (16.0)	41 (11.4)	18 (7.4)
College and higher	57 (8.0)	90 (18.5)	44 (12.4)	58 (23.8)	13 (3.6)	32 (13.3)
None	47 (6.6)	55 (11.4)	13 (3.6)	44 (18.0)	34 (9.5)	11 (4.5)
Ethnicity						
Dalit	209 (29.2)	132 (27.2)	111 (31.2)	71 (29.1)	98 (27.3)	61 (25.2)
Janajati	2 (0.3)	13 (2.7)	1 (0.3)	5 (2.1)	1 (0.3)	8 (3.3)
Brahmin, Chhetri, Thakuri	498 (69.7)	341 (70.1)	238 (66.8)	168 (68.8)	260 (72.4)	173 (71.5)
Other	6 (0.8)	0 (0.0)	6 (1.7)	0 (0.0)	0 (0.0)	0 (0.0)
Total people living in the household						
1–5 people	189 (26.4)	256 (52.7)	120 (33.7)	149 (61.1)	69 (19.2)	107 (44.2)
6–10 people	479 (67.0)	218 (44.9)	219 (61.5)	87 (35.7)	260 (72.4)	131 (54.2)
11–15 people	44 (6.1)	11 (2.3)	15 (4.2)	7 (2.9)	29 (8.1)	4 (1.6)
> 16 people	3 (0.5)	1 (0.1)	2 (0.6)	1 (0.3)	1 (0.3)	0 (0.0)
Electricity connection in the household						
Yes	144 (20.1)	130 (26.7)	129 (36.2)	111 (45.5)	15 (4.2)	19 (7.8)
No	571 (79.9)	356 (73.3)	227 (63.8)	133 (54.5)	344 (95.8)	223 (92.2)
Number of rooms in the household						
One	557 (77.9)	395 (80.9)	225 (63.2)	165 (67.6)	332 (92.5)	230 (94.3)
Two	138 (19.3)	82 (16.8)	111 (31.2)	69 (28.3)	27 (7.5)	13 (5.3)
Three	13 (1.8)	11 (2.3)	13 (3.6)	10 (4.1)	0 (0.0)	1 (0.4)
Four	7 (1.0)	0 (0.0)	7 (2.0)	0 (0.0)	0 (0.0)	0 (0.0)
Number of children in the household						
One	230 (32.2)	153 (31.5)	127 (35.7)	87 (35.7)	103 (28.7)	66 (27.3)
Two	302 (42.2)	173 (35.6)	158 (44.4)	85 (34.8)	144 (40.1)	88 (36.4)
Three	142 (19.9)	147 (30.3)	60 (16.8)	64 (26.2)	82 (22.8)	83 (34.2)
Four	33 (4.6)	11 (2.3)	8 (2.2)	6 (2.5)	25 (7.0)	5 (2.1)
Five	5 (0.7)	2 (0.3)	2 (0.6)	2 (0.8)	3 (0.8)	0 (0.0)
Six	3 (0.4)	0 (0.0)	1 (0.3)	0 (0.0)	2 (0.6)	0 (0.0)
Children below age of 5						
None	179 (25.0)	248 (51.0)	96 (26.9)	118 (48.4)	83 (23.1)	130 (53.7)
One	374 (52.3)	186 (38.3)	200 (56.2)	92 (37.7)	174 (48.5)	94 (38.9)
Two	152 (21.3)	48 (9.9)	58 (16.3)	31 (12.7)	94 (26.2)	17 (7.0)
Three	7 (1.0)	4 (0.8)	1 (0.3)	3 (1.2)	6 (1.7)	1 (0.4)
Four	2 (0.3)	0 (0.0)	1 (0.3)	0 (0.0)	1 (0.3)	0 (0.0)
Five	1 (0.1)	0 (0.0)	0 (0.0)	0 (0.0)	1 (0.2)	0 (0.0)
Sex of the children included into the study						
Male	318 (44.5)	222 (45.7)	146 (41.0)	98 (40.2)	172 (47.9)	124 (51.2)
Female	397 (55.5)	264 (54.3)	210 (59.0)	146 (59.8)	187 (52.1)	118 (48.8)
Age of the children included into the study						
6–60 months	479 (67.0)	74 (15.2)	232 (65.2)	37 (15.2)	247 (68.8)	37 (15.3)
> 61 months	236 (33.0)	412 (84.8)	124 (34.8)	207 (84.8)	112 (31.2)	205 (84.7)

*Note: 715 children were surveyed during the baseline survey. Among those, 486 could be identified and reassessed during the endline survey.*

### COVID-19 and knowledge about and application of preventive measures

The caregivers’ responses to questions about COVID-19 knowledge, attitude, and practices, preventative WASH practices, and the application of other preventive measures are presented in Table 2. More than half of the respondents reported COVID-19 cases in their family, with 1 to 5 individuals per household (72.9%) having been infected, and 12.0% reported that family members died of COVID-19. Almost 94.2% of the respondents mentioned that they were aware of COVID-19 and that major symptoms of COVID-19 include fever (83.3%) and cough (79.4%). The majority (87.8%) of the respondents reported having received some special information on particular hygiene measures to avoid COVID-19, such as social distancing (60.5%) and wearing face masks in public (52.9%). However, during our survey, none of the respondents were seen with face masks, nor were they observed complying with any social distancing protocols.

**Table 2:** COVID-19 related information among surveyed households in Dailekh and Achham districts, December 2021

COVID-19 related information	[N (%)]	Dailekh [n (%)]	Acham [n (%)]	*P*-value
Heard about COVID-19	457 (94.2)	230 (94.6)	227 (93.8)	0.69
Knowledge about symptoms of COVID-19				
Fever	405 (83.3)	196 (80.3)	209 (86.4)	0.07
Cough	386 (79.4)	189 (77.5)	197 (81.4)	0.28
Head pain	232 (47.7)	140 (57.4)	92 (38.0)	0.01
Difficulty of breathing	158 (32.5)	86 (35.2)	72 (29.7)	0.20
Loss of taste	116 (23.9)	45 (18.4)	71 (29.3)	0.01
Pain in the throat	65 (13.4)	39 (16.0)	26 (10.7)	0.09
Loss of smell	61 (12.6)	22 (9.0)	39 (16.1)	0.02
Pain in the joints	50 (10.3)	32 (13.1)	18 (7.4)	0.04
Tiredness	20 (4.1)	16 (6.6)	4 (1.7)	0.01
Diarrhea	14 (2.9)	9 (3.7)	5 (2.1)	0.29
Conjunctivitis	10 (2.1)	5 (2.0)	5 (2.1)	0.99
Received any special information on particular hygiene measures that should be taken to avoid COVID-19 during the pandemic	402 (87.8)	198 (85.7)	204 (89.9)	0.17
Self-reported preventive measures taken after receiving information on COVID-19				
Social distancing	294 (60.5)	150 (61.5)	144 (59.5)	0.66
Wear face mask in public	257 (52.9)	132 (54.1)	125 (51.7)	0.59
Wash hands more often	196 (40.3)	86 (35.3)	110 (45.5)	0.02
Constructed handwashing station	127 (26.1)	85 (34.8)	42 (17.4)	0.01
Regularly disinfect drinking water	127 (26.1)	59 (24.2)	68 (28.1)	0.33
Disinfect hands regularly	92 (18.9)	48 (19.7)	44 (18.2)	0.68
Routes of transmitting the virus				
By breathing aerosols from infected persons	299 (61.5)	166 (68.0)	133 (55.0)	0.01
If virus comes in contact with mucus membranes after touching contaminated surfaces	236 (48.6)	105 (43.0)	131 (54.1)	0.01
By drinking contaminated water	81 (16.7)	55 (22.5)	26 (10.7)	0.01
Other routes mentioned	2 (0.4)	2 (0.8)	0 (0.0)	0.16
Measures used to prevent the spread of COVID-19				
Wear face masks	411 (84.6)	203 (83.2)	208 (86.0)	0.40
Keep social distance	389 (80.0)	200 (82.0)	189 (78.1)	0.29
Regularly wash hands	374 (77.0)	181 (74.2)	193 (79.7)	0.15
Disinfect contaminated surfaces (or hands)	60 (12.4)	32 (13.1)	28 (11.6)	0.61
None	2 (0.4)	2 (0.8)	0 (0.0)	0.16
Other protection mentioned	0 (0.0)	0 (0.0)	0 (0.0)	0.00
Application of above mentioned protective measures				
Never	40 (8.7)	30 (13.0)	10 (4.4)	0.01
Seldom	70 (15.3)	14 (6.1)	56 (24.7)	
Sometimes	211 (46.2)	99 (43.0)	112 (49.3)	
Often	128 (28.0)	79 (34.3)	49 (21.6)	
Always	8 (1.8)	8 (3.5)	0 (0.0)	
At least one family member contracted COVID-19	268 (58.8)	150 (65.2)	118 (52.2)	0.01
Number of family members that contracted COVID-19				
None	1 (0.4)	0 (0.0)	1 (0.8)	0.56
1–5	194 (72.9)	109 (73.1)	85 (72.7)	
6–10	70 (26.3)	39 (26.2)	31 (26.5)	
11–15	1 (0.4)	1 (0.7)	0 (0.0)	
Number of family members with a confirmed diagnosis of COVID-19				
None	192 (42.0)	82 (35.5)	110 (48.6)	0.02
1–5	256 (56.0)	144 (62.3)	112 (49.6)	
6–10	9 (2.0)	5 (2.2)	4 (1.8)	
Measures taken for treating COVID-19 infected persons				
Stay at home and treat with herbal medicine	257 (52.9)	142 (58.2)	115 (47.5)	0.02
Stay at home and treat with medication from pharmacy	20 (4.1)	12 (4.9)	8 (3.3)	0.37
Go to the health center for a test	15 (3.1)	9 (3.7)	6 (2.5)	0.44
Go to the hospital	8 (1.6)	6 (2.5)	2 (0.8)	0.16
Number of family members needing treatment in the health center or hospital due to COVID-19				
None	153 (42.3)	5 (3.4)	148 (69.5)	0.01
One	106 (29.3)	52 (34.9)	54 (25.3)	
Two	85 (23.5)	76 (51.0)	9 (4.2)	
Three	15 (4.1)	15 (10.1)	0 (0.0)	
Four	3 (0.8)	1 (0.7)	2 (0.9)	
Family members dying due to COVID-19	43 (11.9)	20 (13.4)	23 (10.8)	0.45
Measures taken to protect family members or the community after having had contact with an infected person				
Stayed in quarantine	282 (61.8)	145 (63.0)	137 (60.6)	0.59
Other measures mentioned	174 (38.2)	85 (37.0)	89 (39.4)	
Information source used for learning about COVID-19				
Radio	316 (65.0)	142 (58.2)	174 (71.9)	0.01
Community members	286 (58.8)	148 (60.7)	138 (57.0)	0.42
Health staff	203 (41.8)	96 (39.3)	107 (44.2)	0.28
Family members	154 (31.7)	82 (33.6)	72 (29.8)	0.36
Television	86 (17.7)	63 (25.8)	23 (9.5)	0.01
Internet	85 (17.5)	64 (26.2)	21 (8.7)	0.01
Public posters	56 (11.5)	28 (11.5)	28 (11.6)	0.97
Newspaper	44 (9.0)	36 (14.8)	8 (3.3)	0.01
Other	0 (0.0)	0 (0.0)	0 (0.0)	0.00
Information source that provided the most helpful information				
Radio	211 (46.2)	79 (34.2)	132 (58.4)	0.01
Community members	120 (26.3)	66 (28.6)	54 (23.9)	
TV	47 (10.3)	37 (16.0)	10 (4.4)	
Health staff	32 (7.0)	15 (6.7)	17 (7.5)	
Family members	24 (5.2)	16 (6.9)	8 (3.5)	
Internet	19 (4.2)	17 (7.4)	2 (0.9)	
Newspaper	2 (0.4)	1 (0.4)	1 (0.4)	
Public posters	1 (0.2)	0 (0.0)	1 (0.4)	
Other	1 (0.2)	0 (0.0)	1 (0.4)	
Community complied with social distancing measures				
To a great extent	25 (5.5)	19 (8.2)	6 (2.6)	0.01
To a moderate extent	89 (19.4)	62 (26.8)	27 (11.9)	
To some extent	146 (31.9)	97 (42.0)	49 (21.6)	
To a small extent	173 (37.8)	42 (18.2)	131 (37.8)	
Not at all	25 (5.5)	11 (4.8)	14 (6.2)	
Top concerns relating to COVID-19				
Contracting COVID-19	231 (47.5)	116 (47.5)	115 (47.5)	0.99
Loss of job	198 (40.7)	109 (44.7)	89 (36.8)	0.08
Friends or family contracting COVID-19	194 (39.9)	89 (36.5)	105 (43.4)	0.12
Mental health or wellbeing	134 (27.6)	79 (32.4)	55 (22.7)	0.02
Loss of income	127 (26.1)	70 (28.7)	57 (23.6)	0.20
Being unable to access healthcare services	85 (17.5)	33 (13.5)	52 (21.5)	0.02
Long-term economic decline	78 (16.0)	24 (9.8)	54 (22.3)	0.01
Being unable to access social services	46 (9.5)	24 (9.8)	22 (9.1)	0.78
No concerns	30 (6.2)	14 (5.7)	16 (6.6)	0.69
Lack of social interaction	18 (3.7)	10 (4.10)	8 (3.3)	0.64
Lack of safety while staying at home	14 (2.9)	12 (4.9)	2 (0.8)	0.01
Increase in cost/availability of goods	12 (2.5)	8 (3.3)	4 (1.7)	0.25
Increased instances of violence	3 (0.6)	2 (0.8)	1 (0.4)	0.57
Other	0 (0.0)	0 (0.0)	0 (0.0)	0.00

*p*-values were obtained by χ2 test

In our study, 61.5% of the respondents stated that breathing aerosols from an infected person is the major route of virus transmission, followed by the virus coming in contact with mucus membranes after touching contaminated surfaces (48.6%) and by drinking contaminated water (16.7%). Notably, wearing face masks (84.6%), maintaining social distancing (80.0%), and regularly washing hands (77.0%) were reported by most of the respondents as measures for preventing COVID-19. About half of the respondents (52.9%) reported staying at home and using herbal medicines to treat COVID-19-infected individuals. Staying in quarantine (61.8%) was the measure most commonly reported to be taken to protect family members and the community after contact with an infected person. Importantly, radio (65.0%) and community members (58.8%) were mentioned as the most helpful sources of information on COVID-19. However, most of the respondents reported compliance with social distancing measures only to a small extent (37.8%). Contracting COVID-19 (47.5%) and job loss (40.7%) were the respondents’ most pressing concerns.

### Changes in water quality, water accessibility, sanitation and hygiene

It is crucial to follow WASH practices to protect human health during outbreaks of infectious diseases, including COVID-19 [60, 62, 63]. Strengthening water security is necessary for preventing and combating such outbreaks because sufficient safe water is needed for drinking and maintaining good hygiene including adequate handwashing [64]. Our findings show that most of the respondents depended on a piped water supply system with communal taps in the village (96.4% at baseline and 61.3% at endline). A majority of the respondents reported a duration of 6 to 30 min for a return trip to the main drinking water source, including time required for queuing and filling containers. Access to a piped household connection increased (1.8% at baseline and 19.7% at endline). Respondents’ knowledge about water purification methods improved between baseline and endline from boiling (51.6–84.6% at endline) to use of filters (48.0–50.8% at endline). The proportion of respondents without knowledge of any method decreased from 42.8% at baseline to 7.8% at endline. Respondents who provided a good explanation of at least three methods of water treatment methods increased from baseline to endline (16.9–61.4%). At endline, water quality was better than at baseline, with a decreased median value of colony forming unit (CFU) of *E.coli* both at the point of collection (12 vs. 29 CFU *E.coli*/100 mL; IQR = 7–83 at baseline and 4–101 at endline) and point of consumption (34 vs. 51.5 CFU *E.coli*/100 mL; IQR = 17–165 at baseline and 8–194 at endline). The water quality improved in both Dailekh and Achham districts. The details of changes in the water quality at the point of collection and point of consumption are presented in Table 3.

**Table 3:** Water supply, water handling, water treatment and water quality at surveyed households before and during the COVID-19 pandemic

Variables	[N (%)]		*P*-value*	Dailekh [n (%)]		*P*-value*	Achham [n (%)]		*P*-value*	Change from baseline to End-line (95% CI)	*P-value*
	Baseline [n (%)]	End-line [n (%)]		Baseline [n (%)]	End-line [n (%)]		Baseline [n (%)]	End-line [n (%)]			
Involvement into water supply system in the community	712 (99.6)	183 (37.6)	0.17	354 (99.4)	133 (54.5)	0.56	358 (99.7)	118 (48.8)	0.21	n/a	n/a
**Main drinking water source** ^**a**^											
Piped water in the house or yard	13 (1.8)	96 (19.7)	0.53	9 (2.5)	40 (16.4)	0.10	4 (1.1)	56 (23.1)	0.46	3.52 (2.00-5.04)	0.01
Piped water in the village	689 (96.4)	298 (61.3)		340 (95.5)	156 (63.9)		349 (97.2)	142 (58.7)			
Rainwater harvesting	0 (0.0)	0 (0.0)		0 (0.0)	0 (0.0)		0 (0.0)	0 (0.0)			
Open source (dug well, pond, spring)	5 (0.7)	39 (8.0)		5 (1.4)	16 (6.6)		0 (0.0)	23 (9.5)			
Protected source (well, spring)	6 (0.8)	51 (10.5)		0 (0.0)	30 (12.3)		6 (1.7)	21 (8.7)			
Unmanaged piped system	2 (0.3)	1 (0.2)		2 (0.6)	1 (0.4)		0 (0.0)	0 (0.0)			
River, stream or canal	0 (0.0)	1 (0.2)		0 (0.0)	1 (0.4)		0 (0.0)	0 (0.0)			
Lake	0 (0.0)	0 (0.0)		0 (0.0)	0 (0.0)		0 (0.0)	0 (0.0)			
Bottled water	0 (0.0)	0 (0.0)		0 (0.0)	0 (0.0)		0 (0.0)	0 (0.0)			
System level chlorination	n/a	28 (5.8)	n/a	n/a	24 (9.8)	0.01	n/a	4 (1.6)	0.01	n/a	n/a
Frequency of scheme level treatment in two years ^a^											
Never	n/a	5 (17.9)	n/a	n/a	5 (20.8)	n/a	n/a	0 (0.0)		n/a	n/a
Seldom		8 (28.6)			8 (33.3)			0 (0.0)			
Sometimes		0 (0.0)			0 (0.0)			0 (0.0)			
Often		12 (42.9)			9 (37.5)			3 (75.0)			
Always		3 (10.7)			2 (8.3)			1 (25.0)			
Time required for trip (back and forth) to main drinking water source, including time required to queue to fill the containers^a^											
< 5 min	108 (15.1)	35 (7.2)	0.68	45 (12.6)	28 (11.5)	0.60	63 (17.6)	7 (2.9)	0.01	-0.25 (-0.96-0.46)	0.49
6–30 min	569 (79.6)	333 (68.5)		285 (80.1)	201 (82.4)		284 (79.1)	132 (54.5)			
31–60 min	34 (4.8)	45 (9.3)		22 (6.2)	15 (6.2)		12 (3.3)	30 (12.4)			
> 61 min	4 (0.6)	73 (15.0)		4 (1.12)	0 (0.0)		0 (0.0)	73 (30.2)			
**Functioning of main drinking water source** ^**a**^											
Yes, functioning well	0 (0.0)	6 (1.2)	0.71	0 (0.0)	5 (2.0)	0.01	0 (0.0)	1 (0.4)	0.01	1.86 (1.23–2.49)	0.01
Yes, functioning but not regularly	665 (93.0)	87 (17.9)		338 (94.9)	42 (17.2)		327 (91.1)	45 (18.6)			
No, not functioning	50 (7.0)	393 (80.9)		18 (5.1)	197 (80.7)		32 (8.9)	196 (80.9)			
Main drinking water source not functional for more than a week during the past 6 months^b^	1 (0.1)	94 (19.3)	0.04	0 (0.0)	31 (12.7)	0.32	1 (0.3)	63 (26.0)	0.01	0.008 (0.001–0.54)	0.03
Knowlede about water treatment methods^b^											
Boiling	369 (51.6)	411 (84.6)	0.13	220 (61.8)	195 (79.9)	0.01	149 (41.5)	216 (89.3)	0.004	1.21 (0.10-14.08)	0.88
Use of filter	343 (48.0)	247 (50.8)	0.01	209 (58.7)	138 (56.6)	0.01	134 (37.3)	109 (45.0)	0.01	0.91 (0.14–5.74)	0.92
Chlorination	48 (6.7)	18 (3.7)	0.51	32 (9.0)	15 (6.2)	0.02	16 (4.5)	3 (1.2)	0.01	1.96 (0.02-199.59)	0.78
Sodis	26 (3.6)	34 (7.0)	0.01	16 (4.5)	23 (9.4)	0.22	10 (2.8)	11 (4.5)	0.03	n/a	n/a
Flocculation and sedimentation	0 (0.0)	34 (7.0)	n/a	0 (0.0)	17 (6.9)	0.98	0 (0.0)	17 (7.0)	0	n/a	n/a
Other	1 (0.1)	1 (0.2)	n/a	1 (0.3)	1 (0.4)	0.31	0 (0.0)	0 (0.0)	0.32	n/a	n/a
Do not know any	306 (42.8)	38 (7.8)	0.57	111 (31.2)	16 (6.6)	0.01	195 (54.3)	22 (9.1)	0.30	1.32 (0.66–2.61)	0.42
**Explanation of the procedures of different water treatment methods** ^**a**^											
Good explanation of at least 4 methods	18 (4.4)	17 (3.7)	0.34	11 (4.5)	7 (3.1)	0.01	7 (4.3)	10 (4.3)	0.01	3.59 (2.57–4.62)	0.01
Good explanation of 3 methods	69 (16.9)	283 (61.4)		40 (16.3)	119 (52.2)		29 (17.7)	164 (70.4)			
Good explanation of 2 methods	133 (32.5)	120 (26.0)		83 (33.9)	74 (32.5)		50 (30.5)	46 (19.7)			
Satisfactory explanation of 1 method	156 (38.1)	40 (8.7)		94 (38.4)	27 (11.8)		62 (37.8)	13 (5.6)			
Cannot explain well	33 (8.1)	1 (0.2)		17 (6.9)	1 (0.4)		16 (9.8)	0 (0.0)			
Treating drinking water^b^	60 (8.4)	252 (51.8)	0.05	55 (15.4)	88 (36.1)	0.01	354 (98.6)	164 (67.8)	0.01	0.001 (0.0001-0.02)	0.01
**Method used to treat drinking water in last two weeks** ^**b**^											
Boiling	5 (0.7)	206 (42.4)	0.48	3 (0.8)	64 (26.2)	0.65	2 (0.6)	142 (58.7)	0.01	0.002 (0.0002-0.03)	0.01
Use of filter	56 (7.8)	66 (13.6)	0.01	52 (14.6)	45 (18.4)	0.01	4 (1.1)	21 (8.7)	0.002	0.34 (0.006–19.01)	0.60
Flocculation and sedimentation	0 (0.0)	10 (2.1)	n/a	0 (0.0)	1 (0.4)	0.01	0 (0.0)	9 (3.7)	0.01	n/a	n/a
Chlorination	0 (0.0)	5 (1.0)	n/a	0 (0.0)	0 (0.0)	0	0 (0.0)	5 (2.1)	0.02	n/a	n/a
Sodis	0 (0.0)	5 (1.0)	n/a	0 (0.0)	0 (0.0)	0	0 (0.0)	5 (2.1)	0.02	n/a	n/a
Drinking water quality at point of collection											
*Escherichia coli* ^*a*^											
0 CFU/100 mL	48 (6.8)	60 (12.5)	0.01	46 (13.0)	46 (18.9)	0.02	2 (0.6)	14 (5.9)	0.30	3.20 (-667.0-673.41)	0.01
1–10 CFU/100 mL	153 (21.6)	160 (33.3)		76 (21.5)	77 (31.7)		77 (21.6)	83 (34.9)			
10–100 CFU/100 mL	346 (48.8)	143 (29.7)		139 (39.4)	69 (28.4)		207 (58.2)	74 (31.1)			
100–1000 CFU/100 mL	144 (20.3)	66 (13.7)		87 (24.6)	34 (14.0)		57 (16.0)	32 (13.4)			
> 1000 CFU/100 mL	18 (2.5)	52 (10.8)		5 (1.4)	17 (7.0)		13 (3.6)	35 (14.7)			
Median and quartile range (Q1/ Q3)	29 (7/ 83)	12 (4/101)		25 (3/106)	9 (1/ 67)		31 (10/64)	17 (5/117)			
Drinking water quality at point of use											
*Escherichia coli* ^*a,c*^											
0 CFU/100 mL	15 (2.1)	35 (7.3)	0.01	13 (3.7)	33 (13.6)	0.01	2 (0.6)	2 (0.8)	0.32	0.85 (0.96–1.74)	0.01
1–10 CFU/100 mL	100 (14.2)	92 (19.1)		59 (16.8)	54 (22.2)		41 (11.7)	38 (16.0)			
10–100 CFU/100 mL	347 (49.4)	194 (40.3)		151 (42.9)	82 (33.7)		196 (56.0)	112 (47.1)			
100–1000 CFU/100 mL	205 (29.2)	106 (22.0)		115 (32.7)	55 (22.6)		90 (25.7)	51 (21.4)			
> 1000 CFU/100 mL	35 (5.0)	54 (11.2)		14 (4.0)	19 (7.8)		21 (6.0)	35 (14.7)			
Median and quartile range (Q1/ Q3)	51.5 (17/ 165)	34 (8/194)		50.5 (12.5/170.5)	21 (3/ 160)		53.5 (23/143)	44.5 (14/ 208)			

^*a*^ *Changes are estimated by mixed linear regression models for the respective end-line outcome, including the random intercepts of the study wards, while also adjusting for the outcomes observed at the baseline, the district, sex and age of the child, and education level and socioeconomic status of the caregivers. The effect estimates can be interpreted as adjusted difference in the mean change of the respective variable between the baseline and endline.*

^*b*^ *Changes in the binary outcomes were calculated using mixed logistic regression models, including the random intercepts for the study wards while also adjusting for the outcomes observed at baseline, the district, sex, and age of the child, and education level and socio-economic status of the caregivers.*

^*c*^ *Colony forming unit*

*P-values were calculated by Wilcoxon rank-sum test for continuous variables and McNemar Test for binary outcomes

Handwashing with soap is a hygiene practice commonly promoted to protect from infectious pathogens [60, 65, 66] and reduce the burden of infectious diseases, including COVID-19 [21, 60, 67]. Over 94.2% of the respondents self-reported washing their hands with soap 5 to 10 times per day during endline compared to 19.6% at baseline, especially after defecation (74.3%) and before meals (67.4%). The number of households with handwashing facilities in the category of high level of hygiene increased from 32.3% [60, 65, 66] at baseline to 46.5%. Beyond handwashing, a clean household environment is crucial to reduce the spread of infectious pathogens, including COVID-19 [68, 69]. Around 73.8% of the households at baseline and 74.3% of the households at endline used a simple pit latrine. A small number of households (7.6% at baseline and 12.1% at endline) used water-sealed latrines. Even though access to improved sanitation facilities is vital to reduce the spread of infectious pathogens, including COVID-19 [70], only a small number of latrines were in the high level of hygiene category (21.2% at baseline and 18.1% at endline). The personal hygiene of participating children and their caregivers improved from baseline to endline, and the number of households in the category of high hygiene increased from 28.9 to 34.4% (Table 4).

**Table 4:** Hygiene behavior and hygiene condition before and during the COVID-19 pandemic

Variables	[N (%)]		*P*-value*	Dailekh [n (%)]		*P*-value*	Achham [n (%)]		*P*-value*	Change from baseline to endline Beta/OR (95% CI)	*P-value*
	Baseline [n (%)]	Endline [n (%)]		Baseline [n (%)]	Endline [n (%)]		Baseline [n (%)]	Endline [n (%)]			
Cleans container for drinking water transport	707 (98.9)	464 (95.5)	0.86	355 (99.7)	238 (97.4)	0.03	352 (98.1)	226 (93.4)	0.03	n/a	n/a
Cleans container for drinking water transport^a^											
Everyday	3 (0.4)	2 (0.4)	0.68	2 (0.6)	2 (0.8)	0.08	1 (0.3)	0 (0.0)	0.04	0.13 (0.05–0.32)	0.16
Every second day	8 (1.1)	66 (14.2)		4 (1.1)	39 (16.4)		4 (1.1)	27 (11.9)			
At least once per week	103 (14.6)	95 (20.5)		40 (11.3)	52 (21.8)		63 (17.9)	43 (19.0)			
Less than once per week	593 (83.9)	301 (64.9)		309 (87.0)	145 (60.9)		284 (80.7)	156 (69.0)			
**Methods of cleaning the container used for transport of water** ^**a**^											
I use water or water and sand	167 (23.6)	280 (60.3)	0.86	37 (10.4)	131 (55.0)	0.01	130 (36.9)	149 (65.9)	0.01	2.09 (-0.01-4.21)	0.05
I use chlorine to disinfect it almost always	0 (0.0)	1 (0.2)		0 (0.0)	1 (0.4)		0 (0.0)	0 (0.0)			
I use chlorine to disinfect it sometime	0 (0.0)	1 (0.2)		0 (0.0)	1(0.4)		0 (0.0)	0 (0.0)			
I wash it almost always with soap or ash	204 (28.8)	132 (28.4)		108 (30.4)	70 (29.4)		96 (27.3)	62 (27.4)			
I wash it sometimes with soap or ash	336 (47.5)	50 (10.8)		210 (59.2)	35 (14.7)		126 (35.8)	15 (6.6)			
Daily frequency of handwashing with soap or ash^a^											
1–4 times	574 (80.3)	26 (5.3)	0.01	278 (78.1)	10 (4.1)	0.17	296 (82.5)	16 (6.6)	0.24	1.92 (1.71–2.14)	0.01
5–10 times	140 (19.6)	458 (94.2)		78 (21.9)	233 (95.5)		62 (17.3)	225 (93.0)			
11–15 times	1 (0.1)	2 (0.4)		0 (0.0)	1 (0.4)		1 (0.3)	1 (0.4)			
≥ 16 times	0 (0.0)	0 (0.0)		0 (0.0)	0 (0.0)		0 (0.0)	0 (0.0)			
Handwashing^b^											
After going to toilet	705 (98.6)	361 (74.3)	0.77	354 (99.4)	194 (79.5)	0.06	351 (97.8)	167 (69.0)	0.008	0.64 (0.02–16.37)	0.79
Before eating	482 (67.4)	482 (67.4)	0.38	187 (52.1)	194 (79.5)	0.01	295 (82.9)	183 (75.6)	0.31	0.08 (0.05–0.12)	0.01
After cleaning baby’s bottom	440 (61.5)	110 (22.6)	0.09	236 (66.3)	58 (23.8)	0.009	204 (56.8)	52 (21.5)	0.55	0.11 (0.01–1.09)	0.06
When they look dirty	385 (53.9)	237 (48.8)	0.25	172 (48.3)	120 (49.2)	0.003	213 (59.3)	117 (48.4)	0.85	1.62 (0.09–28.74)	0.74
Before cooking	256 (35.8)	164 (33.7)	0.98	142 (39.9)	104 (42.6)	0.02	114 (31.7)	60 (24.8)	0.01	0.29 (0.02–3.58)	0.34
There are no special occasions	1 (0.1)	1 (0.2)	n/a	0 (0.0)	1 (0.4)	0.32	1 (0.3)	0 (0.0)	0.32	n/a	n/a
Never	2 (0.3)	0 (0.0)	n/a	2 (0.6)	0 (0.0)	n/a	0 (0.0)	0 (0.0)	n/a	n/a	n/a
Defecation^a^											
Using bushes	4 (0.6)	7 (1.4)	0.21	3 (0.8)	2 (0.8)	0.54	1 (0.3)	1 (0.4)	0.24	1.72 (0.89–2.56)	0.01
A shared simple pit latrine	56 (7.8)	17 (3.5)		12 (3.4)	9 (3.7)		44 (12.3)	31 (12.8)			
A shared water sealed toilet	73 (10.2)	42 (8.6)		61 (17.1)	50 (20.5)		12 (3.3)	2 (0.8)			
Households own simple pit latrine	528 (73.8)	361 (74.3)		263 (73.9)	171 (70.1)		265 (73.8)	184 (76.0)			
Households own water sealed toilet	54 (7.6)	59 (12.1)		17 (4.8)	12 (4.9)		37 (10.3)	24 (9.9)			
Hygiene condition of latrine^a,c^											
Low hygiene category	279 (42.3)	162 (33.3)	0.14	17 (7.0)	91 (37.3)	0.01	71 (29.3)	71 (29.3)	0	0.94 (0.86–1.02)	0.01
Intermediate hygiene category	240 (36.4)	236 (48.6)		136 (55.7)	136 (55.7)		100 (41.3)	100(41.3)			
High hygiene category	140 (21.2)	88 (18.1)		91 (37.3)	17 (7.0)		71 (29.3)	71 (29.3)			
Hygiene condition of the handwashing facilities^a,d^											
Low hygiene category	54 (40.6)	154 (31.7)	0.02	43 (43.9)	74 (30.3)	0.04	11 (31.4)	80 (33.1)	0.46	-0.06 (-1.53-1.42)	0.94
Intermediate hygiene category	36 (27.1)	106 (21.8)		29 (29.6)	67 (27.5)		7 (20.0)	39 (16.1)			
High hygiene category	43 (32.3)	226 (46.5)		26 (26.5)	103 (42.2)		17 (48.6)	123 (50.8)			
Hygiene condition of kitchen^a,e^											
Low hygiene category	256 (35.8)	93 (19.1)	0.01	151 (42.4)	39 (16.0)	0.01	105 (29.2)	54 (22.3)	0.14	0.89 (-0.36-2.14)	0.16
Intermediate hygiene category	253 (35.4)	216 (44.4)		130 (36.5)	112 (45.9)		123 (34.3)	104 (43.0)			
High hygiene category	206 (28.8)	177 (36.4)		75 (21.1)	93 (38.1)		131 (36.5)	84 (34.7)			
Personal hygiene of participating child and their caregivers^a,f^											
Low hygiene category	273 (38.2)	147 (30.2)	0.01	186 (52.2)	49 (20.1)	0.01	87 (24.2)	98 (40.5)	0.01	0.07 (-0.08-0.22)	0.35
Intermediate hygiene category	235 (32.9)	172 (35.4)		111 (31.2)	111 (45.5)		124 (34.5)	61 (25.2)			
High hygiene category	207 (28.9)	167 (34.4)		59 (16.6)	84 (34.4)		148 (41.2)	83 (34.3)			
Information received on water, sanitation and hygiene^b^	37 (5.2)	307 (63.2)	0.28	19 (5.3)	134 (54.9)	0.84	18 (5.0)	173 (71.5)	0.01	0.03 (0.005–1.93)	0.10

^*a*^ *Changes were assessed by mixed linear models for the respective end-line outcome, including the random intercepts of the study wards, while also adjusting for the outcomes observed at the baseline, the district, sex and age of the child, and education level and socioeconomic status of the caregivers. The effect estimates can be interpreted as adjusted difference in the mean change of the respective variable between the baseline and endline.*

^*b*^ *Changes in the binary outcomes were calculated using mixed logistic regression models, including the random intercepts for the study wards while also adjusting for the outcomes observed at baseline, the district, sex, and age of the child, and education level and socio-economic status of caregivers.*

^*c*^ *A new variable for the observed hygiene condition of the latrine was created using factor analysis with four conceptually similar categorical variables: (i) is the toilet clean; (ii) are these materials available (sandals, drum with water, brush, none of these). The condition of toilet was then categorized into three categories with a low, intermediate and high hygiene category.*

^*d*^ *A new variable for the observed hygiene condition of the handwashing facility was created using factor analysis with four conceptually similar categorical variables: (i) are handwashing facilities in good condition; (ii) are handwashing facilities clean; (iii) is soap available; (iv) is water available. The condition of the handwashing facility was then categorised into three categories with a low, intermediate and high hygiene category.*

^*e*^ *A new variable for the observed hygiene of the kitchen hygiene condition was created using factor analysis with for a conceptually similar categorical variables: (i) are clean dishes kept high; (ii) is the entirety of food covered; (iii) is there a rack to dry your utensils and dishes after washing and (iv) is there a significant number of flies in the kitchen (> 10). The kitchen hygiene was then categorised into the categories with lower, middle and better hygiene.*

^*f*^ *A new variable for the observed personal hygiene of the caregiver and the participating child was created using factor analysis with four conceptually similar categorical variables of: (i) wearing shoes; (ii) hands are clean, (iii) piles of dirty clothes lying around in the house. The personal hygiene was then categorised into three categories with lower, intermediate and high hygiene category.*

* P-values were calculated by Wilcoxon rank sum test for continuous variables and McNemar test for binary outcomes

### Changes in child health and intestinal parasitic infections among study children

The change in health and intestinal parasitic infections among children is shown in Table 5. Infectious diseases, such as fever (41.1–16.8%; *p* = 0.01), cough (36.9–14.4%; *p* = 0.01), respiratory illness (14.3–4.3%; *p* = 0.002), and diarrhea (19.6–9.5%; *p* = 0.01) were higher during baseline than at endline in Achham district, and these changes are statistically significant. Blood (5.6–10.7%) and mucus in stool (5.3–17.4%) increased strongly. Similarly, at baseline, the prevalence of intestinal parasitic infections except for *Trichuris trichura* and hookworm, among children in the study areas were all high (20.6% *Ascaris lumbricoides*, 8.0% *Hymenolepsis nana*, 4.6% *Enterobius vermicularis* and 34.2% *Giardia lamblia*). At the endline, intestinal parasitic infection, especially of *Giardia lamblia*, had declined strongly (7.1% *Ascaris lumbricoides*, 1.3% *Hymenolepsis nana*, 1.2% *Enterobius vermicularis* and 6.5% *Giardia lamblia*). The prevalence of hookworm increased from 2.6% at baseline to 11.3% at endline.

**Table 5:** Child health and intestinal parasitic infections before and during the COVID-19 pandemic

Health variables	[N (%)]		*P*-value	Dailekh [n (%)]		*P*-value	Achham [n (%)]		*P*-value	Persistence^a^ of child health problems (95% CI) (OR)^c^	*P-value*	Incidence^b^ of child health problems (95% CI) (OR)^c^	*P-value*
	Baseline [n (%)]	Endline [n (%)]		Baseline [n (%)]	Endline [n (%)]		Baseline [n (%)]	Endline [n (%)]					
Child suffered from illness in past 7 days													
Fever	294 (41.1)	81 (16.8)	0.01	137 (38.5)	27 (11.1)	0.01	157 (43.7)	54 (22.3)	0.01	0.44 (0.11–1.73)	0.24	0.31 (0.11–0.89)	0.03
Cough	264 (36.9)	70 (14.4)	0.01	126 (35.4)	25 (10.2)	0.01	138 (38.4)	45 (18.6)	0.01	0.70 (0.18–2.68)	0.60	0.13 (0.05–0.38)	0.01
Respiratory illness	102 (14.3)	21 (4.3)	0.002	39 (11.0)	5 (2.1)	0.001	63 (17.5)	16 (6.6)	0.01	0.02 (0.01–3.40)	0.14	0.04 (0.07–0.28)	0.01
Diarrhea	140 (19.6)	46 (9.5)	0.01	69 (19.4)	3 (1.2)	0.01	71 (19.8)	43 (17.8)	1.00	0.19 (0.01–3.12)	0.24	0.60 (0.05–0.75)	0.03
Blood in stool	25 (3.5)	28 (5.8)	0.69	5 (1.4)	2 (0.8)	0.41	20 (5.6)	26 (10.7)	0.02	n/a	n/a	0.03 (0.004–0.24)	0.01
Mucus in stool	27 (3.8)	43 (8.8)	0.13	8 (2.3)	1 (0.4)	0.01	19 (5.3)	42 (17.4)	0.32	n/a	n/a	0.06 (0.004–0.84)	0.04
Blood in urine	3 (0.4)	2 (0.4)	0.32	0 (0.0)	1 (0.4)	0.32	3 (0.8)	1 (0.4)	1.00	n/a	n/a	0.01 (0.01–0.43)	0.01
Intestinal parasites													
* Ascaris lumbricoides*	88 (20.6)	34 (7.1)	0.01	68 (38.4)	16 (6.8)	0.01	20 (8.0)	18 (7.9)	0.85	**	**	0.11 (0.02–0.53)	0.01
* Trichuris trichura*	5 (1.2)	8 (1.7)	0.63	3 (1.7)	6 (2.5)	0.32	2 (0.8)	2 (0.9)	1.00	**	**	0.02 (0.08–0.50)	0.02
* Hymenolepsis nana*	34 (8.0)	6 (1.3)	0.45	10 (5.7)	4 (1.7)	0.26	24 (9.6)	1 (0.4)	0.01		**	n/a	n/a
Hookworm	11 (2.6)	55 (11.3)	0.01	8 (4.5)	29 (12.2)	0.004	3 (1.2)	19 (8.3)	0.002	**	**	0.08 (0.02–0.33)	0.01
* Enterobius vermicularis*	19 (4.6)	6 (1.2)	0.18	9 (5.1)	6 (2.5)	0.09	10 (4.0)	0 (0.0)	0.01	**	**	n/a	n/a
* Giardia lamblia*	146 (34.2)	31 (6.5)	0.01	33 (18.6)	16 (6.7)	0.005	113 (45.2)	13 (5.7)	0.01	**	**	0.03 (0.004–0.27)	0.01

^*a*^ *Persistence was analyzed in the sample of children who had the outcome at baseline*

^*b*^ *Incidence among the children was them who were free of outcome at baseline*

^*c*^ *Odds ratios (OR) were calculated using mixed logistic regression models of the outcome at endline as a function of the outcome at baseline, with the random intercept of the study ward and further adjustments for the district, age, sex and educational status of the child, and socioeconomic status of the caregivers*

*P-values were calculated using the McNemar test*

### Changes in dietary diversity, clinical signs of nutritional deficiencies and malnutrition among study children

The details of changes in the outcomes, dietary diversity and signs of nutritional deficiencies and malnutrition, are presented in Tables 6 and 7. All clinical signs of nutritional deficiencies had increased by endline in both districts, especially bitot’s spot (26.7–40.2%; *p* = 0.01), loss of hair pigment (13.3–41.4%; *p* = 0.01), dry and infected cornea (19.4–36.7%; *p* = 0.01), pale conjunctiva (47.0–63.3%; *p* = 0.01), spongy bleeding gums (17.8–66.7%; *p* = 0.01), and dermatitis (64.8–81.4%; *p* = 0.01). The numbers of severely stunted (26.6–48.5%; *p* = 0.01) and underweight (19–54.9%; *p* = 0.01) children had increased at endline. However, thinness decreased (9.2–7.7%; *p* = 0.65) (Table 6).

**Table 6:** Clinical signs of nutritional deficiencies and malnutrition among study children before and during the COVID-19 pandemic

Clinical signs of nutritional deficiency and malnutrition	[N(%)]		P-value*	Dailekh [n (%)]		*P*-value*	Achham [n (%)]		*P*-value*	Persistence^a^ of child health problems (95% CI) (OR)^c^ or (B)^d^	*P-value*	Incidence^b^ of child health problems (95% CI) (OR)^c^ or (B)^d^	*P-value*
	Baseline [n (%)]	Endline [n (%)]		Baseline [n (%)]	Endline [n (%)]		Baseline [n (%)]	Endline [n (%)]					
Bitot’s spot	191 (26.7)	197 (40.2)	0.01	89 (25.0)	98 (40.3)	0.01	102 (28.4)	90 (38.5)	0.005	0.80 (0.23–2.83)	0.73	1.16 (0.59–2.25)	0.67
Loss of hair pigment	95 (13.3)	203 (41.4)	0.01	56 (15.7)	113 (46.5)	0.01	39 (10.9)	83 (35.5)	0.01	1.18 (0.15–9.22)	0.88	0.69 (0.33–1.46)	0.34
Dry and infected cornea	139 (19.4)	180 (36.7)	0.01	49 (13.8)	93 (38.3)	0.01	90 (25.1)	76 (32.5)	0.001	0.47 (0.12–1.83)	0.28	1.39 (0.62–3.14)	0.42
Oedema	29 (4.1)	49(10.0)	0.01	9 (2.5)	21 (8.6)	0.003	20 (5.6)	27 (11.5)	0.004	n/a	n/a	0.10 (0.04–0.28)	0.01
Pale conjunctiva	336 (47.0)	310 (63.3)	0.01	154 (43.3)	157 (64.6)	0.01	182 (50.7)	144 (61.5)	0.004	13.6 (2.42–76.46)	0.00	5.37 (1.70–16.90)	0.01
Bowed legs	27 (3.8)	28 (5.7)	0.12	9 (2.5)	8 (3.3)	0.56	18 (5.0)	18 (7.7)	0.09	n/a	n/a	n/a	n/a
Spongy bleeding gums	127 (17.8)	327 (66.7)	0.01	70 (19.7)	140 (57.6)	0.01	57 (15.9)	178 (76.1)	0.01	3.14 (0.69–14.17)	0.14	3.16 (1.12–8.89)	0.03
Dermatitis	463 (64.8)	399 (81.4)	0.01	235 (66.0)	188 (77.4)	0.002	228 (63.5)	201 (85.9)	0.01	22.14 (5.35–91.56)	0.01	12.39 (3.04–50.57)	0.01
Red inflamed tongue	175 (24.5)	120 (24.5)	0.63	80 (22.5)	53 (21.8)	1.00	95 (26.5)	65 (27.8)	0.42	0.76 (0.24–2.45)	0.65	0.62 (0.28–1.37)	0.24
Sub-dermal hemorrhage	42 (5.9)	107 (21.8)	0.01	19 (5.3)	43 (17.7)	0.01	23 (6.4)	61 (26.1)	0.01	n/a	n/a	0.82 (0.43–1.55)	0.54
Goiter	5 (0.7)	18 (3.7)	0.01	1 (0.3)	12 (4.9)	0.001	4 (1.1)	5 (2.1)	0.03	n/a	n/a	n/a	n/a
Stunting (height for age, HAZ)^d^													
Low	323 (45.2)	115 (33.6)	0.01	161 (45.2)	63 (31.3)	0.01	162 (45.1)	52 (36.9)	0.01	1.41 (0.51–3.92)	0.50	1.91 (0.73–5.01)	0.19
Medium	202 (28.3)	61 (17.8)		98 (27.5)	37 (18.4)		104 (29.0)	24 (17.0)					
Severe	190 (26.6)	166 (48.5)		97 (27.3)	101 (50.3)		93 (25.9)	65 (46.1)					
Underweight (weight for age, WAZ)	136 (19.0)	269 (54.9)	0.01	66 (18.5)	156 (64.2)	0.33	70 (19.5)	101 (43.2)	0.78	3.92 (1.11–13.87)	0.03	3.38 (1.36–8.41)	0.01
Thinness (wasting, body mass index for age, BAZ)	66 (9.2)	37 (7.7)	0.65	35 (9.8)	17 (8.5)	0.24	31 (8.6)	11 (7.8)	0.72	n/a		0.90 (0.03–0.32)	0.01

^*a*^ *Persistence was analyzed in the sample of children who had the outcome at baseline*

^*b*^ *Incidence among the children who were free of outcome at baseline*

^*c*^ *Odds ratios (OR) were calculated using mixed logistic regression models of the outcome at endline as a function of the outcome at baseline, with the random intercept of the study ward and further adjustments for the district, age, sex and educational level of the child, and socioeconomic status of the caregivers*

^*d*^ *B were calculated by mixed linear regression model for the outcome at endline, including the random intercepts of the study wards and further adjustments for the district, sex and age, sex and education level of the child and socioeconomic status of the caregivers*

*P values were calculated using the McNemar test and Wilcoxon-signed rank test

**Table 7:** Food consumption details and food security before and during the COVID-19 pandemic

Food consumption and food security	[N (%)]		*P-*value	Dailekh [n (%)]		*P*-value	Achham [n (%)]		*P*-value	Change in consumption Beta/OR (95% CI)	*P-value*
	Baseline [n (%)]	Endline [n (%)]		Baseline [n (%)]	Endline [n (%)]		Baseline [n (%)]	Endline [n (%)]			
**Food consumption details**											
Number of meals a child eats per day^a^											
One meal	4 (0.6)	0 (0.0)	0.12	0 (0.0)	0 (0.0)	0.06	4 (1.1)	0 (0.0)	0.01	0.13 (0.04–0.23)	0.01
Two meals	15 (2.1)	14 (2.9)		4 (1.1)	9 (3.7)		11 (3.1)	5 (2.1)			
Three meals	441 (61.7)	267 (55.0)		216 (60.7)	89 (36.5)		225 (62.7)	178 (73.9)			
Four meals	222 (31.0)	200 (41.2)		119 (33.4)	144 (59.0)		103 (28.7)	56 (23.2)			
Five meals	31 (4.3)	3 (0.6)		15 (4.2)	2 (0.8)		16 (4.5)	1 (0.4)			
Six meals	2 (0.3)	1 (0.2)		2 (0.6)	0 (0.0)		0 (0.0)	1 (0.4)			
Beans, peas or lentils^a^											
three times per day	14 (2.0)	1 (0.2)	0.01	7 (2.0)	1 (0.4)	0.01	7 (2.0)	0 (0.0)	0.01	6.14 (5.10–7.18)	0.01
twice per day	2 (0.3)	1 (0.2)		1 (0.3)	0 (0.0)		1 (0.3)	1 (0.4)			
once per day	0 (0.0)	8 (1.6)		0 (0.0)	2 (0.8)		0 (0.0)	6 (2.5)			
every second day	7 (1.0)	56 (11.5)		1 (0.3)	18 (7.4)		6 (1.7)	38 (15.7)			
two times per week	12 (1.7)	159 (32.7)		1 (0.3)	59 (24.2)		11 (3.1)	100 (41.3)			
once per week	348 (48.7)	255 (52.5)		147 (41.3)	162 (66.4)		201 (56.0)	93 (38.4)			
less than once per week	319 (44.6)	6 (1.2)		195 (54.8)	2 (0.8)		124 (34.5)	4 (1.7)			
not at all	13 (1.8)	0 (0.0)		4 (1.1)	0 (0.0)		9 (2.5)	0 (0.0)			
Dairy products (milk, yoghurt)^a^											
three times per day	14 (2.0)	10 (2.1)	0.01	7 (2.0)	9 (3.7)	0.12	7 (2.0)	1 (0.4)	0.01	1.23 (-0.58-3.03)	0.18
twice per day	331 (46.3)	68 (14.0)		175 (49.2)	38 (15.6)		156 (43.5)	30 (12.4)			
once per day	49 (6.8)	69 (14.2)		13 (3.6)	41 (16.8)		36 (10.0)	28 (11.6)			
every second day	68 (9.5)	49 (10.1)		42 (11.8)	26 (10.7)		26 (7.2)	23 (9.5)			
two times per week	27 (3.8)	44 (9.0)		17 (4.8)	12 (4.9)		10 (2.8)	32 (13.2)			
once per week	8 (1.1)	166 (34.2)		4 (1.1)	81 (33.2)		4 (1.1)	85 (35.1)			
less than once per week	127 (17.8)	79 (16.3)		65 (18.3)	37 (15.2)		62 (17.3)	42 (17.4)			
not at all	91 (12.7)	1 (0.2)		33 (9.3)	0 (0.0)		58 (16.2)	1 (0.4)			
Meat or fish^a^											
three times per day	5 (0.7)	3 (0.6)	0.01	1 (0.3)	2 (0.8)	0.01	4 (1.1)	1 (0.4)	0.01	2.71 (1.05–4.37)	0.01
twice per day	170 (23.8)	90 (18.5)		53 (14.9)	31 (12.7)		117 (32.6)	59 (24.4)			
once per day	90 (12.6)	189 (38.9)		33 (9.3)	91 (37.3)		57 (15.9)	98 (40.5)			
every second day	302 (42.2)	119 (24.5)		169 (47.5)	72 (29.5)		133 (37.0)	47 (19.4)			
two times per week	60 (8.4)	49 (10.1)		41 (11.5)	31 (12.7)		19 (5.3)	18 (7.4)			
once per week	58 (8.1)	22 (4.5)		32 (9.0)	11 (4.5)		26 (7.2)	11 (4.6)			
less than once per week	25 (3.5)	14 (2.9)		22 (6.2)	6 (2.5)		3 (0.8)	8 (3.3)			
not at all	5 (0.7)	0 (0.0)		5 (1.4)	0 (0.0)		0 (0.0)	0 (0.0)			
Leafy green vegetables^a^											
three times per day	96 (13.4)	28 (5.8)	0.01	78 (21.9)	18 (7.4)	0.01	18 (5.0)	10 (4.1)	0.58	3.57 (1.88–5.26)	0.01
twice per day	4 (0.6)	23 (4.7)		2 (0.6)	14 (5.7)		2 (0.6)	9 (3.7)			
once per day	5 (0.7)	33 (6.8)		4 (1.1)	12 (4.9)		1 (0.3)	21 (8.7)			
every second day	11 (1.5)	65 (13.4)		5 (1.4)	30 (12.3)		6 (1.7)	35 (14.5)			
two times per week	13 (1.8)	170 (35.0)		4 (1.1)	67 (27.5)		9 (2.5)	103 (42.6)			
once per week	419 (58.6)	165 (34.0)		173 (48.6)	101 (41.4)		246 (68.5)	64 (26.5)			
less than once per week	160 (22.4)	2 (0.4)		87 (24.4)	2 (0.8)		73 (20.3)	0 (0.0)			
not at all	7 (1.0)	0 (0.0)		3 (0.8)	0 (0.0)		4 (1.1)	0 (0.0)			
Fruit^a^											
three times per day	34 (4.8)	24 (4.9)	0.01	13 (3.7)	4 (1.6)	0.01	21 (5.9)	20 (8.3)	0.01	3.39 (1.68–5.10)	0.01
twice per day	645 (90.2)	96 (19.7)		314 (88.2)	51 (20.9)		331 (92.2)	45 (18.6)			
once per day	3 (0.4)	109 (22.4)		0 (0.0)	48 (19.7)		3 (0.8)	61 (25.2)			
every second day	11 (1.5)	74 (15.2)		9 (2.5)	38 (15.6)		2 (0.6)	36 (14.9)			
two times per week	6 (0.8)	78 (16.1)		6 (1.7)	36 (14.8)		0 (0.0)	42 (17.4)			
once per week	2 (0.3)	77 (15.8)		2 (0.6)	49 (20.1)		0 (0.0)	28 (11.6)			
less than once per week	9 (1.3)	26 (5.3)		8 (2.2)	16 (6.6)		1 (0.3)	10 (4.1)			
not at all	5 (0.7)	2 (0.4)		4 (1.1)	2 (0.8)		1 (0.3)	0 (0.0)			
Harvest sufficient to meet household’s yearly food requirements^b^											
No	250 (35.0)	458 (94.2)	0.01	96 (27.0)	238 (97.5)	0.01	154 (42.9)	220 (90.9)	0.01	3.11 (0.09-102.59)	0.52
Yes	465 (65.0)	28 (5.8)		260 (73.0)	6 (2.5)		205 (57.1)	22 (9.1)			
Requirement to buy extra food^a^											
Do not produce own food	0 (0.0)	11 (2.4)	0.89	0 (0.0)	1 (0.4)	0.01	0 (0.0)	10 (4.6)	0.30	2.44 (1.29–3.61)	0.01
Upto 3 months	90 (19.4)	142 (31.0)		55 (21.1)	74 (31.1)		35 (17.1)	68 (30.9)			
3 to 6 months	135 (29.0)	61 (13.3)		83 (31.9)	45 (18.9)		52 (25.4)	16 (7.3)			
More than 6 months	200 (43.0)	195 (42.6)		113 (43.5)	105 (44.1)		87 (42.4)	90 (40.9)			
Do not need to buy any food	40 (8.6)	49 (10.7)		9 (3.5)	13 (5.5)		31 (15.1)	36 (16.4)			

^*a*^ *Changes were assessed by mixed linear models for the respective end-line outcome, including the random intercepts of the study wards, while also adjusting for the outcomes observed at the baseline, the district, sex and age of the child, and education level and socioeconomic status of the caregivers. The effect estimates can be interpreted as adjusted difference in the mean change of the respective variable between the baseline and end-line.*

^*b*^ *Changes in the binary outcomes were calculated using mixed logistic regression model, including the random intercepts for the study wards while also adjusting for the outcomes observed at baseline, the district, sex and age of the child, education level and socio-economic status of caregivers.*

^*P value are calculated by Wilcoxon rank sum test*^

The number of children eating up to four meals per day increased from 31.0% at baseline to 41.2% at endline. However, the consumption of dairy products twice per day decreased (46.3–14.0%; *p* = 0.01). Similarly, the consumption of lentils, meat and fish and leafy green vegetables once per week decreased (48.7–52.5%, *p* = 0.01; 8.1–4.5%, *p* = 0.01; and 58.6–34.0% *p* = 0.01, respectively). Overall, food security decreased drastically. At endline, an increased number of households reported that the harvest was inadequate for their households’ yearly nutritional needs (35.0–94.2%; *p* = 0.01) in both Dailekh (27.0–97.5%; *p* = 0.01) and Achham (42.9–90.9%; *p* = 0.01). This could be due to decreased agricultural production and fewer resources available in the household to purchase food due to reduced household income.

### Challenges faced by children due to COVID-19

The qualitative interviews revealed a number of problems faced by children due to COVID-19. The major problems highlighted are as follows:

#### Handwashing

Two respondents reported during in-depth interviews that the lack of soap due to its high cost and the disappearance of soap from handwashing stations are the major causes why their children are not washing their hands with soap. Also, the government never had offered them help with soap.

*We have difficult terrain here at Achham, so we do not have good accessibility to roads. We need to walk at least 4 h to reach Kamalbazar [nearby town] to fulfill our basic needs. Things are expensive these days. It is difficult for us to access even very much needed things like soap and medicines. If soap comes to our village, then the price increases more than double. So, we need to travel ourselves to get it. Unfortunately, these soaps often get lost when we keep them at the handwashing stations.*

-Female respondent, 32–33 years old (November 2021).

*It’s a pity that our village is so much behind. … behind in terms of road, electricity, education, and also water supply. I think government (especially the mayor) has never known that our lives also matter. They never helped us, not even with a single bar of soap during the COVID-19 pandemic. They always disappear after election.*

-Male respondent, 38 years (November 2021).

#### Water access and treatment

At the community level, female caregivers and the village water supply committee members reported challenges with piped water supply services in the communities. They mentioned difficulties with the water connection, leaking problems due to an old pipeline, water not being supplied regularly, and water only being supplied during 60 min per day.

*There is a water system with taps in some communities, however, no regular flow of water in it. Water is available once in the early morning for one hour at maximum and then it stops for 24 h. If you fill up your vessels within this time, you will have adequate water, else you need to wait till the next day. The water may sometimes even be inadequate for our basic hygiene needs.*

-Female caregiver respondent, 26 years old (December 2021).

*Last year, there were around 70 water taps installed in some communities in the village. Unfortunately, these taps are not functioning and water still is scarce [in our village]. Meanwhile, we do not have electricity [in our village]. Hence, we cannot use things like a motor for the village water tank for a regular supply of water to these installed taps. Even in the schools, there is lack of water and water containers, hence students lack water for drinking and washing after defecation. Even during the COVID-19 pandemic, the water supply issues remained the same and were not sorted out.*

-Male community stakeholder, 72 years old (December 2021).

Several respondents pointed out that problems with water availability were not related to COVID-19 and the lockdowns. At least half of the respondents described water scarcity as being a problem mostly during the summer season, due to water sources drying up and piped water systems being destroyed or obstructed during rains.

*Every year we have a problem of the water source getting dry during summer. And there is an issue of landslides during monsoon season and the water pipes from the water source getting buried. During this time of the year, we need to walk a minimum of two hours up to the river to get drinking water.*

-Female caregiver, 29 years old (December 2021).

Some interviewees reported that water treatment filters had been distributed for a subsidized price and that they were not happy about not getting one. However, in a majority of households, black kettles were found to be available for boiling water.

*Only [names] were provided with a [water] filter. We were here and we have not been given any [water filter]. We as neighbors are only seeing them using that [water filter].*

-Female caregiver, 70 years old (December 2021).

### Sanitation

The pandemic and lockdowns did not have any impact on children’s reported toilet use. The villages do not have a proper fecal sludge management system. Although some villages in Achham were declared “open defecation free” villages before the endline survey, human feces could still be seen around the walking paths. No public latrines were found in the study villages.

### Socioeconomic status

About 70.0% of the respondents’ husbands had been working in India as daily wage laborers. Due to the COVID-19 pandemic and lockdowns, the husbands returned home and thus could no longer contribute to household incomes. This led to widespread financial insecurity in the state. Because the agricultural lands are not fertile enough and lack water for irrigation, the loss in household income could not be compensated by increasing agricultural production. This added to the risk of insufficient food security for many families.

*Suddenly a lockdown was declared in India and he [husband] came home. We don’t know until when it will last. We don’t have another source of income. He is the only person earning [for us] and we are 8 people here [in home]. For doing agriculture, there is no good water supply system in this village, and the agricultural lands are not fertile enough.*

-Female caregivers, 29 years old (December 2020).

### Child education

The caregivers informed that the children were not able to go to school due to COVID-19 and the lockdown since early 2020. The village did not have access to electricity or the internet for online teaching.

*For almost two years the children are here [home] like this. Schools are closed. Teachers are relaxing. They [children] play, eat, sleep… it is like that. I think even we [parents] forgot already that they need to go [to school] [laughing]. They cannot study watching television or mobile as we do not have electricity here in our village. After two to three years, they will also join their father in India.*

-Female caregiver, 60 years old (December 2020)

## Discussion

We found the COVID-19 pandemic increased vulnerability, reduced income, aggravated already high poverty levels and increased already severe nutritional deficiencies in our study areas. In contrast, hygiene behavior such as the frequency of handwashing improved. The mortality rate due to COVID-19 was relatively high in the study areas we surveyed and was similar to other parts of the country [71]. It is likely that the high mortality rates due to COVID-19 in Dailekh and particularly in Achham were associated with weak and inadequate WASH infrastructure, inadequate local public health infrastructure, and the government’s limited efforts, capacities, and expertise to chart any emergency measures or to set up health care facilities for treating COVID-19 patients [9, 72]. Our study revealed that COVID-19-infected individuals mostly stayed at home and used herbal medicines for treatment. Other studies indicated that in addition to the lack of accessibility and availability of health care resources, social stigma related to COVID-19 contributed to a low number of patients visiting health facilities for treatment [73, 74]. Moreover, the long-term closure of transportation services due to the lockdown might have been one of the major factors hindering access to health services, which might have added to the extreme hardship among the respondents [9, 35, 75].

Our findings showed that the majority of the respondents received information on hygiene measures to avoid COVID-19 such as handwashing and disinfecting drinking water and hands regularly. The female community health volunteers played a crucial role in supporting community health care, especially when the majority of health facilities were closed or disrupted, as was also reported in Province 2 of Nepal [74]. The most frequently mentioned preventative measures were to wear face masks, maintain social distancing, and wash hands regularly. However, our research assistants observed that following these preventive measures was problematic. A majority of the households had an average of 6 to 10 people living together in one room. This made adherence to social distancing protocols impossible. Practicing social distancing in a low-income setting, where many people live in very dense small spaces, is also an enormous challenge in other parts of the world [11, 76–78]. Furthermore, many families had at least one person who returned home from India during our study, but the quarantine protocols were generally not followed by the returning migrants. During our survey, the community members did not use any face masks, gloves, or hand sanitizers when interacting with each other. The cost and affordability of the disinfecting materials may be the reason behind this [60].

We found that in our study areas, significant improvements in field hygiene practices had taken place. The frequency of handwashing with soap or ash significantly increased, by a factor of about two, despite people complaining about the high cost of soap and the difficulty of obtaining it due the interruption of supply chains. However, other WASH conditions are still inadequate and constitute a risk of transmission of infectious diseases. The people in the study areas still practice open defecation and have poor sanitation infrastructure with ineffective fecal sludge management systems. Water supply is irregular, and although water quality improved both at the source and at the point of consumption, a substantial proportion of water samples were still contaminated.

Our study found reduced intestinal parasitic infections, except for hookworm, and a reduced incidence of self-reported infectious diseases, such as fever, cough, respiratory illnesses, and diarrhea, among the children surveyed despite the COVID-19 pandemic and lockdowns. These health improvements may be associated with hygiene improvements, particularly handwashing, after the promotion of hygiene measures to avoid COVID-19 [79, 80].

Despite the reduction of infectious diseases, including diarrhea and parasitic infections, which may be associated with children’s improved nutritional status [81, 82], the already critical nutritional status of the children surveyed further declined between baseline and endline with an increase in both clinical signs of malnutrition and severe stunting and underweight. This concerning development is very likely linked with a reduction in the quantity and quality of food provided to children during the COVID-19 pandemic. The economic consequences of the lockdowns on child health were severe, especially among those already struggling with the adverse impacts of poverty and hunger [83]. Historically, the children of the study areas have long faced food and nutrition security challenges with a high prevalence of malnutrition [9, 44, 46, 84]. The COVID-19 pandemic exacerbated this and led to a loss of wages that reduced the resources available for purchasing food, thus putting this vulnerable population, especially children, at risk of hunger and malnutrition. Similar findings have been reported in other rural parts of Nepal [9, 85].

Despite significant improvements in hygiene behavior, the WASH conditions in the study area need further improvement to reduce the risk of transmitting pathogens via the fecal–oral route and prevent future disease outbreaks. Adequate and effective WASH measures are crucial for public health [1]. Extensive collaboration between organizations supporting WASH improvements and public health organizations are recommended to improve effective governance, management, and communication strategies in the WASH sector [1, 22]. In addition, future campaigns for the prevention of disease outbreaks need to consider and mitigate the potential negative impact of measures such as lockdowns that prevent income-generating activities. The findings of our study indicated that economic challenges among particularly marginalized families could be associated with very concerning negative impacts on child health.

Our study has some limitations: First, all the responses are self-reported. This could have introduced a reporting bias, as respondents sometimes may have over-reported hygienic behaviors. Furthermore, self-reports of more frequent handwashing do not indicate whether handwashing has been performed correctly. Second, we could not capture the detailed impacts of the COVID-19 pandemic in these extremely hard-to-reach areas of Nepal. Third, this study was conducted in the Dailekh and Achham districts of Nepal. These areas are characterized by extremely poor health status indicators. Hence, the results may not be generalizable to other areas of Nepal. Fourth, due to this study’s nature, it is impossible to infer a causal association between WASH practices and child health outcomes such as the decrease in intestinal infection. Fifth, a large number of households migrated away from the study area. Therefore, only 69.0% of children could be re-assessed during the endline survey.

Despite the limitations, our study has several strengths. First, we used mixed methods to triangulate between the WASH practices and problems before and during the COVID-19 pandemic and changes in the children’s health and nutritional status. Second, we had baseline data available from the pre-COVID-19 period. This gave us the unique opportunity to document changes in the health status of children, WASH practices, and nutrition before and during the pandemic. Thus, our study contributes to documenting the multifaceted impacts of the COVID-19 pandemic on a remote rural community and sheds some light on areas that may require particular attention to mitigate the negative effects of future epidemics, such as improving access to health services and hygiene materials and carefully considering the considerable economic and social impacts of lockdowns.

## Conclusion

In our study, we documented water management, sanitation, hygiene practices, nutrition provided to the children and child health before and during the COVID-19 pandemic in very remote hilly areas of Nepal, which are characterized by a lack of infrastructure, such as roads, electricity, limited health services and lacking access to digital and electronic communication channels. We found that during the pandemic, the frequency of handwashing increased significantly. At the same time, infectious diseases such as fever, cough, respiratory illnesses, diarrhea, and intestinal parasitic infections decreased significantly, indicating that there could be an association between the uptake of improved WASH behavior during the pandemic and a reduction of the risk of infectious disease. The local community received information about measures to prevent COVID-19 mostly via traditional channels such as the radio and community health workers and most frequently rated the wearing of face masks, social distancing, and regular handwashing as protective measures. Contrary to handwashing, the wearing of face masks and social distancing was not practiced consistently due to limited availability, the high cost of face masks, and the lack of space in households. Access to soap and adequate water was also found to be challenging. The economic consequences of the lockdowns and associated restrictions were quite severe: most respondents reported reduced employment and daily income and, therefore, reduced access to nutrition. It seems likely that the decline in access to nutrition in these already food-stressed areas was associated with the increase in observed in nutritional deficiencies among the children surveyed. Our findings highlights that disaster preparedness should pay more attention to ensuring access to materials required for adequate hygiene practices, such as water and soap for handwashing and other protective materials. Our findings also show that measures to combat an epidemic in remote regions can be two sided: on one side, people can be motivated to improve hygiene practices. This could contribute to improve protection against infectious diseases. On the other side, lockdowns are very problematic for low-income households and are associated with negative economic, social, and health consequences.

## Electronic supplementary material

Below is the link to the electronic supplementary material.

Supplementary Material 1

Supplementary Material 2

## Data Availability

The dataset and the questionnaire supporting the conclusions are available from the corresponding author on reasonable request.

## List of abbreviations

LMICs: low- and middle- income countries
SDG: Sustainable Development Goals
WASH: water, sanitation and hygiene
WHO: World Health Organization
